# ‘I thought I had fibroids, and now I don’t’: a mixed method study on health-related quality of life in uterine sarcoma patients

**DOI:** 10.1186/s12955-022-01971-5

**Published:** 2022-04-20

**Authors:** Dide den Hollander, Emma Lidington, Susanne Singer, Samantha C. Sodergren, Samer Salah, Marco Fiore, Charlotte Benson, Ingrid M. E. Desar, Vivian W. G. Burgers, Olga Husson, Winette T. A. van der Graaf

**Affiliations:** 1 Department of Medical Oncology, Netherlands Cancer Institute, Antoni Van Leeuwenhoek, Amsterdam, The Netherlands; 2 Department of Medical Oncology, Radboud University Medical Center, Nijmegen, The Netherlands; 3 Sarcoma Unit, The Royal Marsden NHS Foundation Trust, London, UK; 4 Institute of Medical Biostatistics, Epidemiology and Informatics, University Medical Centre Mainz, Mainz, Germany; 5 University Cancer Centre Mainz (UCT), University Medical Centre Mainz, Mainz, Germany; 6 School of Health Sciences, University of Southampton, Southampton, UK; 7 Department of Medical Oncology, King Hussein Cancer Center, Amman, Jordan; 8 Department of Surgery, Fondazione IRCCS Istituto Nazionale Dei Tumori, Milan, Italy; 9 Division of Clinical Studies, Institute of Cancer Research, London, UK; 10 Department of Medical Oncology, Erasmus MC Cancer Institute, Erasmus University Medical Center, Rotterdam, The Netherlands

**Keywords:** Soft tissue sarcoma, Uterine sarcoma, Gynaecological sarcoma, Health-related quality of life, Patient-reported outcomes

## Abstract

**Background:**

Uterine sarcomas are rare subtypes of primary urogenital tumours and need tailored treatment. This study aimed to examine the impact of diagnosis and treatment on health-related quality of life (HRQoL) in patients with uterine sarcoma and measures available to assess HRQoL in this group.

**Methods:**

Thirteen patients with uterine sarcoma and 23 health care professionals were purposively sampled from sarcoma reference centers and participated in a semi-structured interview exploring HRQoL. Patients were also asked to review the EORTC QLQ-C30 and EORTC QLQ-EN24 for relevance. Data were analysed using thematic analysis and descriptive statistics.

**Results:**

The most commonly reported physical health issues were related to sexual dysfunction and urological symptoms. Hormone-related issues and gastrointestinal symptoms were also identified. Cancer-generic issues such as functional problems, fatigue, pain, and treatment-related adverse effects were also reported. Regarding mental health, fears (about having sex, of recurrence, or of death), altered body-image, and dealing with lacking knowledge regarding sarcoma had an impact on HRQoL. Social health issues were related to the impact on relationships with others, limitations in undertaking activities, loss of independence, changes in work or study capacity, and financial difficulties. Most of the items of the EORTC QLQ-C30 and EORTC QLQ-EN24 questionnaires were rated as relevant. Questions about lack of knowledge about sarcoma, shock of diagnosis, and menopausal symptoms were lacking from existing measures.

**Conclusions:**

Uterine sarcoma patients experience a variety of concerns covering the physical, mental, and social domains of HRQoL that are in the main EORTC instruments, but not all of them. Combining cancer-generic, location- and sarcoma-specific items is recommended to assess HRQoL in this patient group.

*Trial registration* NCT04071704.

**Supplementary Information:**

The online version contains supplementary material available at 10.1186/s12955-022-01971-5.

## Introduction

Being diagnosed with and treated for sarcoma has a significant impact on health-related quality of life (HRQoL) [1, 2]. However, few studies have investigated HRQoL outcomes in patients with uterine soft tissue sarcoma (uterine STS) specifically. Assessment of HRQoL in this rare subgroup of sarcomas is difficult because tailored instruments for HRQoL measurement are currently not available. Exploration of the HRQoL issues that patients with uterine STS experience and assessing content validity of existing patient-reported outcome measures (PROMs) will help increase knowledge of HRQoL outcomes in this patient group.

STS account for only a small proportion of primary urogenital tumours, i.e. 3% of all uterine malignancies. Data on urogenital sarcomas as a group are hardly available and limited survival rates are reported for these rare sarcoma locations, with the exception of uterine sarcoma. In this group, survival greatly varies depending on histological subtypes with uterine leiomyosarcoma that has a 5-year relative survival across all stages of 42%, compared to 72% for endometrial stromal tumours [3].

Tailored treatment is very important in STS and differs from treatment for endometrial cancer, which occurs at the same anatomical location. The cornerstone of treatment for localized uterine STS is en bloc total hysterectomy. A survival benefit of bilateral salpingo-oophorectomy has not been demonstrated especially not in pre-menopausal women. Routine lymph node dissection is not indicated [4]. Adjuvant chemotherapy or radiation therapy have not been proven to increase survival rates, but may be considered in selected cases [5, 6]. Adjuvant hormonal therapy might be considered in patients with low grade, estrogen/progesterone receptor positive endometrial stromal sarcomas [7]. In metastatic uterine sarcoma, doxorubicin remains standard first line treatment. However, first-line combination chemotherapy with gemcitabine and docetaxel can be considered as well in advanced uterine leiomyosarcoma patients [8]. In selected cases of metastatic hormone receptor positive ESS and uterine leiomyosarcomas, hormonal therapy is an option [7, 9].

In addition to objective treatment outcomes, such as survival rates, subjective patient-reported outcomes (PROs) including health-related quality of life (HRQoL) can be used to assess treatment effectiveness. HRQoL is a multidimensional concept that includes the patient’s perception of the impact of the disease and its treatment on physical/biological (including symptoms), psychological and social functioning, according to the biopsychosocial model [10, 11].

An instrument to measure HRQoL in uterine STS patients during and after treatment does currently not exist, although some symptoms were included in a non-validated sarcoma-specific symptom-inventory [12]. The EORTC Quality of Life Core Questionnaire 30 (EORTC QLQ-C30) [13] combined with the EORTC QLQ-Endometrial Cancer Module (EORTC QLQ-EN24) [14] are validated to assess HRQoL in women treated for endometrial cancer, however it is unknown if this measurement strategy is applicable for the uterine STS population.

One cohort study has previously investigated HRQoL, using the EORTC Quality of Life Core Questionnaire 30 (EORTC QLQ-C30), and long-term treatment effects in a heterogeneous group of bone and soft tissue sarcoma survivors. Fifteen female urogenital sarcoma survivors were included, with over 40% reporting pain in the back, urge incontinence, flatulence, abdominal cramps, bloated feeling of the abdomen, and complaints similar to menopause [15]. Two studies have investigated patient-reported symptoms at time of diagnosis of uterine sarcoma, being heavy menstrual flow, painful menstruation, “persistent abdominal pain that is getting worse” and “fibroids or lumps that are increasing in size” [12, 16]. Nevertheless, information about the psychosocial impact of the disease and treatment is lacking.

The current mixed-method study aimed to gain more insight in the HRQoL issues that patients with uterine STS face and how this impact can be measured, to improve sensitive HRQoL assessment in research and to further optimize personalized care in this rare cancer type.

## Methods

### Design

This inductive analysis is part of a larger mixed method study with a convergent design to determine a strategy for HRQoL measurement in all types of adult sarcomas [17] (clinicaltrials.gov NCT04071704), conducted in multiple countries in Europe and beyond. Outside Europe, patients were recruited from Australia, Canada, Hong Kong, India, Israel, Jordan, and the United States. The qualitative approach was chosen to perform a phenomenological inquiry of HRQoL issues experienced by uterine sarcoma patients. Subsequently, the quantitative approach was used to assess whether existing questionnaires for patients with cancer at the same anatomical location (i.e. endometrial cancer) can be used to assess HRQoL in this patient group rather than creating a completely new item list.

### Sample and procedure

Inclusion and exclusion criteria and procedure for recruitment for this study have been described elsewhere [17]. Recruitment continued until saturation was reached for the sarcoma population as a whole in the larger study. A stratification matrix was used to ensure that the heterogeneity of the sarcoma population in terms of tumour location, histological subtypes, disease stage (localized, metastatic disease/ local relapse), treatments, age, sex, and physical impairments was adequately covered. At least 12 patients per location were recruited. For this analysis, patients with primary sarcoma in the uterus were selected.

Furthermore, international sarcoma specialists were invited and interviewed by the study coordinator or the local principal investigator. The sarcoma specialists worked as medical oncologists, radiation therapists, surgeons, nurse specialists, rehabilitation physicians, psychologists, or social workers and are hereafter referred to as health care professionals (HCPs). For this analysis, only HCPs who had experience with uterine STS patients were selected.

All patients gave written informed consent prior to the interview. Sociodemographic data were collected from the medical file or obtained from the patient directly at study entry. Clinical data was also collected from the medical file by a member of the study team.

Ethical approval was given by the Institutional Review Board of the Netherlands Cancer Institute (IRB18121). In the other international participating centres, this study was approved by their ethical committees according to local regulations.

### Interviews

Semi-structured interviews were conducted between July 2019 and July 2020, by a trained physician, psychologist, researcher, or research nurse (DdH, EL, SSi, SSo, SSa, MF, VB), using an interview guide. Participants were asked an open-ended question about their cancer history and experience and were then prompted according to the interview guide provided to all collaborators (Additional file 1: File 1). Subsequently, the participant was asked to review (i.e. not complete) the EORTC QLQ-C30 and EORTC QLQ-EN24, and comment on topic and item relevance with regard to the period from diagnosis up to time of this study (scored as 1—“not all relevant”, 2—“a little relevant”, 3—“quite a bit relevant” and 4—“very much relevant”).

The participant was encouraged to “think aloud”, to provide feedback on the reasons for his/her ratings. Finally, the participants were asked whether items could be deleted or other HRQoL issues that they believe to be important could be added to the questionnaires. Given the large sample size and heterogeneity of the population in the large mixed method study, member checking was not done. Reflexivity was only addressed through the interpretations by the different international interviewers speaking with sarcoma patients and HCPs.

### Questionnaires

The EORTC QLQ-C30 version 3.0 was developed to measure HRQoL in patients with cancer [13]. This 30-item questionnaire consists of five functional scales (physical, role, cognitive, emotional and social), a global quality of life scale, three symptom scales (fatigue, pain, nausea and vomiting) and single items assessing common symptoms (dyspnoea, loss of appetite, sleep disturbance, constipation, and diarrhoea) and perceived financial impact of the disease. Cronbach’s alpha coefficients range from 0.52 to 0.89 after treatment (physical functioning 0.71, role functioning 0.52, cognitive functioning 0.73, emotional functioning 0.80, social functioning 0.77, global QoL 0.89, fatigue 0.85, nausea and vomiting 0.73, pain 0.76). Scaling successes were noted in 96% and al inter-scale correlations were statistically significant.

The EORTC QLQ-EN24 was developed for HRQoL measurement in patients with endometrial cancer and consists of 24 items [14]. It measures five symptom scales (lymphedema, urological symptoms, gastrointestinal symptoms, poor body image and sexual/vaginal problems), five single symptoms items (pain in back and pelvis, tingling/numbness, muscular pain, hair loss and taste change) and three functional single items (sexual interest, sexual activity and sexual enjoyment). Cronbach’s alpha coefficients range from 0.74 to 0.86 (lymphoedema 0.80, urological symptoms 0.75, gastrointestinal symptoms 0.74, body image problems 0.86 and sexual/vaginal problems 0.86) and convergent and discriminant validity has no scaling errors for the subscales.

### Data analysis and reporting

Interviews were audio-recorded, transcribed verbatim, translated and checked for accuracy by the local research team to prevent loss of context. Transcripts of interviews were anonymized before they were sent to the coders. Two coders (DdH and MR) performed thematic analysis on the data in NVIVO version 12. Transcripts of interviews were examined in detail in order to identify basic patterns and recurrent themes using line-by-line coding [18, 19]. The biopsychosocial model was the framework in which key themes and subthemes were created by comparing each theme with the rest of the data to create analytical categories and then grouping categories together [11]. Coders discussed their findings, adjusted themes, and resolved differences until consensus was reached. Themes were created for uterine STS patients and HCPs separately, but sequentially by the same two coders. Thus, coding for HCPs was informed by previous coding from the patient group. This created uniformity of themes within the biopsychosocial model for patients and HCPs to facilitate the development of a measurement strategy for HRQoL in uterine sarcoma patients. Translations of quotes selected for this manuscript were checked by a native English speaker. The COnsolidated criteria for REporting Qualitative research (COREQ) for this study are reported in Additional file 2: File 2.

Data from the questionnaire reviews (comment on topic and item relevance with regard to the period from diagnosis up to time of this study) were analysed using descriptive statistics (e.g. missing data, means, standard deviations) and prevalence ratio (number of patients who experienced each complaint, i.e. who scored 2, 3 or 4, divided by the total number of patients who completed that item, multiplied by 100). As an exploratory aim, relevance of the items in these questionnaires was analysed with cut-off values adapted from the decision rules for selection of items in phase 1 studies in the EORTC Quality of Life Group Guidelines for Developing Questionnaire modules [20], also described in a separate publication of the study protocol [17]:

- Mean score > 1.5
- Prevalence ratio > 30%

If relevance scores for an item met both the aforementioned criteria, the item was considered relevant.

## Results

### Participants

Thirteen patients with uterine STS participated in semi-structured interviews to gain insight in the HRQoL issues they face and how this impact can be measured. Additionally, 23 HCPs with experience of working with uterine STS patients commented on issues specifically relevant in this patient group. Sociodemographic and clinical information of patients and HCPs is presented in Table 1.

**Table 1:** Sociodemographic and clinical characteristics of study participants

	Uterine sarcoma (N = 13)
*Sociodemographic and characteristics patients*	
Mean age at time of diagnosis (SD, range)	50.8 (7.3, 38–61) Missing n = 1
Mean age at time of study (SD, range)	53.3 (6.4, 39–61) Missing n = 1
Country	
Germany	2
Italy	1
Jordan	1
The Netherlands	4
United Kingdom	5
Relationship status	
Single	2 (15.4%)
Married	8 (61.5%)
Separated/divorced	2 (15.4%)
Widowed	1 (7.7%)
Living arrangement	
Alone	3 (23.1%)
With partner	3 (23.1%)
With children 18 years or older	1 (7.7%)
With partner and children	5 (38.5%)
Missing	1 (7.7%)
Highest formal education	
Primary school only	1 (7.7%)
High school	3 (23.1%)
College or university	9 (69.2%)
Employment status	
Full-time	2 (15.4%)
Part-time	3 (23.1%)
Unemployed	1 (7.7%)
Home maker	1 (7.7%)
Student	1 (7.7%)
Retired	3 (23.1%)
Sick leave	2 (15.4%)
Clinical characteristics	
Histology	
Endometrial stromal sarcoma	2 (15.4%)
Leiomyosarcoma	6 (46.2%)
Rhabdomyosarcoma	1 (7.7%)
Sarcoma NOS	1 (7.7%)
Other*	3 (23.1%)
Stage at diagnosis	
Localized	10 (76.9%)
Metastatic	3 (23.1%)
Treatment at diagnosis	
Chemotherapy	4
Radiotherapy	2
Hormonal therapy	1
Surgery	12
Stage at study enrolment	
Localized	3 (23.1%)
Metastatic	7 (53.8%)
Follow-up	3 (23.1%)
*HCP characteristics*	
Sex	
Male	13 (56.5%)
Female	10 (43.5%)
Mean age (SD, range)	47 (7.7, 33–60) (1 missing)
Country	
Canada	2 (8.7%)
Cyprus	1 (4.3%)
Germany	2 (8.7%)
Italy	1 (4.3%)
Jordan	2 (8.7%)
Spain	2 (8.7%)
The Netherlands	7 (30.4%)
United Kingdom	6 (26.1%)
Profession	
Medical oncologist	10 (43.5%)
Nurse specialist	3 (13%)
Physiotherapist	1 (4.3%)
Psychologist	1 (4.3%)
Radiation oncologist	2 (8.7%)
Surgeon	4 (17.4%)
Palliative care consultant	1 (4.3%)
Clinical oncologist (UK)	1 (4.3%)
Mean years of experience in profession (SD, range)	14.5 (8.4, 3–32) (2 missing)
Mean years of experience in sarcoma care (SD, range)	12.7 (7.0, 5–27) (3 missing)

*1 adenosarcoma, 1 PEComa, 1 undifferentiated spindle cell sarcoma

### Interview analysis-main themes

From the thematic analyses of the patient and HCP interviews, a total of eighteen, eleven, and six subthemes were defined within the physical, mental, and social health themes, respectively. The issues from the subthemes were further divided into categories, which are presented side by side for patients and HCPs in Table 2. HCPs reported both issues specifically for uterine STS patients only and issues relevant to the sarcoma patients’ population in general (indicated in Table 2). All related quotes from the interviews are presented in Additional file 1: File 3.

**Table 2:** Representation of themes from interviews and coverage by EORTC QLQ-C30 and EORTC EN-24

Patient	HCP		
*Phyiscal health*			
1. Gastrointestinal symptoms	1. Gastrointestinal symptoms	EORTC QLQ-C30	EORTC QLQ-EN24
1.1 Bloated feeling of the abdomen			X
1.2 Pressure in the abdomen	1.2 Heaviness in the abdomen^a^		
1.3 Feeling a mass in the abdomen	1.3 Swelling of the abdomen^a^		
1.4 Bowel movement problems	1.4 Problems with defecation		
1.4.1 Constipation	1.4.1 Constipation	X	
1.4.2 Diarrhoea	1.4.2 Diarrhoea	X	
1.4.3 Tingling sensation in hips when I need to open my bowels			
1.5 Lack of appetite	1.5 Lack of appetite^b^	X	
1.6 Gastro-esofageal reflux			
1.7 Nausea/ vomiting	1.7 Nausea/ vomiting^b^	X	
1.8 Abdominal pain	1.8 Abdominal pain		
	1.9 Rectal bleeding^a^		
2. Sexual problems	2. Sexual problems		
2.1 Decreased ability to reach an orgasm			
2.2 Decreased interest in sex			X
2.3 Less sexual enjoyment			X
2.4 Vaginal dryness	2.4 Vaginal dryness^a^		X
	2.5 Vagina feels short or tight^a^		X
	2.6 Pain during sex		X
3. Gynaecological symptoms	3. Gynaecological symptoms		
3.1 Heavy periods	3.1 Heavy periods^a^		
3.2 Abnormal vaginal bleeding	3.2 Abnormal vaginal bleeding^a^		
	3.3 Vaginal discharge^a^		
4. Urinary problems	4. Urinary problems		
4.1 Needed a catheter/ catheterising			
4.2 Urinary incontinence	4.2 Urgency to go to the toilet to urinate		X
4.3 Urinating frequently			X
	4.4 Pain when passing urine		X
5. Hormonal problems	5. Hormonal problems		
	5.1 Menopausal symptoms (“hot flushes, night sweats, insomnia, anxiety, sexual problems, vaginal dryness”)		
5.2 Experiencing menopausal symptoms because I cannot take hormone-replacement therapy	5.2 No treatments possible for menopausal symptoms		
	5.3 Going into menopauze early		
	5.4 Pain in muscles and joints		X
6. Neurological symptoms	6. Neurological symptoms		
6.1 Numbness	6.1 Numbness^b^		X
6.2 Tingling	6.2 Tingling in feet		X
7. Functional impairment	7. Functional impairment		
7.1 Impairments in ADL	7.1 Impairment in ADL (Needed help with ADL)	X	
7.2 Impairments in mobility	7.2 Impairment in mobility		
7.3 Physical impairment	7.3 Physical impairment^b^	X	
8. Lack of energy	8. Lack of energy		
8.1 Feeling tired	8.1 Feeling tired^b^	X	
8.2 Need to rest		X	
8.3 Feeling weak and lacking energy	8.3 Feeling weak and lacking energy^b^	X	
9. Trouble sleeping	9. Trouble sleeping^b^	X	
9.1 Trouble sleeping because of having to go to bathroom frequently			
9.2 Trouble sleeping because of worries			
10. Pain	10. Pain	X	
10.1 Circumstances of pain			
10.1.1 Postoperative pain			
10.1.2 Pain when lifting heavy things			
10.2 Different locations of pain			
10.2.1 Pain of the lump			
10.2.2 Pain in area of surgery			
10.2.3 Back pain			X
10.3 Need to take pain medication	10.3 Need to take pain medication^b^		
11. Radiotherapy related problems	11. Radiotherapy related problems^b^		
11.1 Burning and painful skin	11.1 Burning and painful skin^b^		
11.2 Blisters of skin			
11.3 Ulcer near the anus			
12. Chemotherapy related issues	12. Chemotherapy related issues^b^		
12.1 Feeling ill	12.1 Feeling ill^b^		
12.2 Lower resistance to infection	12.2 Lower resistance to infection^b^		
12.3 Hair problems	12.3 Hair problems^b^		X
12.4 Nail problems			
12.5 Eye problems			
12.6 Mouth problems	12.6 Mouth problems^b^		
12.7 Nose problems			
12.8 Skin problems	12.8 Skin problems^b^		
13. Weight problems	13.Weight problems		
13.1 Weight gain	13.1 Weight gain		
13.2 Weight loss	13.2 Weight loss		
14. Respiratory problems	14. Respiratory problems		
14.1 Shortness of breath	14.1 Shortness of breath^b^	X	
14.2 Trouble breathing on exertion			
15. Symptoms related to metastasis or local treatment for metastasis			
15.1 Eating problems			
15.2 Mouth problems			
15.3 Problems with speaking			
	16. Local effects of tumour		
	16.1 Fistulation^a^		
	16.2 Mass protruding from vagina^a^		
	16.3 Deep venous thrombosis		
	16.4 Leg problems		X
17. Surgery effects	17. Surgery effects		
	17.1 Herniation		
17.2 Improvement of quality of life after treatment	17.2 Improvement of quality of life after treatment		
	17.3 Nephrostomy		
	17.4 Swelling of legs		X
	18. Infertility		
*Mental health*			
1.Fears/Worries	1. Anxiety/Worries	X	
1.1 Fear of recurrence			
1.1.1 Scanxiety	1.1.1 Scanxiety^b^		
1.1.2 Fear of recurrence	1.1.2 Fear of recurrence^b^		
	1.1.3 Anxiety due to delay of diagnosis and treatment		
	1.1.4 Anxiety after initial misdiagnosis		
1.2 Fear of death	1.2 Fear of death^b^		
1.3 Fear for treatment			
1.4 Fears about having sex			
1.5 Worried about your condition’s impact on loved ones	1.5 Worried about patient’s condition’s impact on loved ones^b^		
2. Dislike of changed appearance/Changed body image	2. Changed body image		
2.1 Hair loss			X
	2.2 Scars^b^		
2.3 Physical deformity	2.3 Physical deformity^b^		
2.4 Felt less attractive	2.4 Felt less attractive		X
	2.5 Felt less feminine		X
	2.6 Less self-esteem		
	2.7 Felt body is changing		
3. Felt constantly reminded about your condition			
4. Living with uncertainty	4. Living with uncertainty^b^		
4.1 Uncertainty regarding treatment			
4.2 Uncertainty regarding the future	4.2 Uncertainty regarding the future^b^		
5. Felt distressed by the lack of knowledge about sarcoma	5. Felt distressed by the lack of knowledge about sarcoma^b^		
6. Desire to have contact with fellow patients	6. Desire to have contact with fellow patients^b^		
7. Change in emotions	7. Change in emotions		
7.1 Feeling sad	7.1 Feeling sad^b^		
7.2 Being more emotional			
8. Felt stigmatized	8. Felt stigmatized^b^		
9. Shock of diagnosis	9. Shock of diagnosis		
	9.1 Sarcoma diagnosis comes unexpectedly after initial misdiagnosis		
	10. Dealing with (risk of) infertility		
	11. Lack of psychosexual support		
	11.1 Sexual problems are rarely discussed spontaneously		
*Social health*			
1. Loss of independence	1. Loss of independence^b^	X	
2. Relationship with others	2. Relationship with others	X	
2.1 Losing contact with friends or family	2.1 Losing contact with friends or family^b^		
2.2 Isolation	2.2 Isolation ^b^		
2.3 Lack of understanding from others	2.3 Lack of understanding from others^b^		
3. Limitation in activities	3. Limitation in activities		
3.1 Limitation in social activities	3.1 Limitation in social activities ^b^	X	
3.2 Limitation in leisure activities or hobbies	3.2 Limitation in leisure activities or hobbies^b^	X	
3.3 Difficulty doing activities with (grand) children			
4. Financial difficulties because of medical costs and medical aid	4. Financial difficulties because of loss of income & medical costs^b^	X	
5. Changes in work or study capacity	5. Changes in work		
5.1 Had to change function at work	5.1 Change in career		
5.2 Limitations in work/study	5.2 Limitations in work	X	
5.3 Not being able to work	5.3 Not being able to work^b^		
6. Having a changed life	6. Having a changed life^b^		

*ADL* activities of daily living

^a^Only reported for uterine sarcoma patients

^b^Reported for sarcoma patient population as a whole

#### Physical health

Uterine tumours caused heavy periods or postmenopausal blood loss, leading to the eventual sarcoma diagnosis. HCPs reported symptoms of vaginal discharge, fistulation, or, in exceptional cases, a protruding mass from the vagina as a direct consequence of the tumour.

Patients and HCPs mentioned that uterine STS also caused gastrointestinal complaints, such as changed bowel habits like constipation or diarrhoea, “*feeling bloated and uncomfortable due to the pressure on my abdomen”* (1pt 3, 61 years, undifferentiated spindle cell sarcoma) or they noticed a growing mass in the abdomen *“.. meanwhile my belly was growing enormously. I even said to a colleague: ‘It seems like I am four months pregnant’.”* (pt 9, 59 years, leiomyosarcoma). The large tumour impacted appetite, leading to weight loss. Treatment with radiotherapy or chemotherapy also caused gastrointestinal symptoms, such as diarrhoea, gastro-esophageal reflux, nausea and vomiting, or rectal bleeding as mentioned by a HCP. After surgery, one patient mentioned she experienced a tingling sensation in her hips when she needed to open her bowels.

Both patients and HCPs reported that after surgery urinary incontinence is common, with leakage of urine and having to rush to the toilet. Patients also had to urinate more frequently, which could be related to the tumour pushing on the bladder or to surgery. Finally, HCPs also reported patients can experience pain when urinating: *“have you had pain or burning feeling when passing urine, yes a lot of them they come with this complaint”* (HCP 2, age missing, medical oncologist).

Being treated for uterine STS had a significant impact on the sexual life of patients. Women described the decreased ability to reach an orgasm after removal of the uterus: *“My orgasm really is much less.’ Because the uterus is a muscle. I had a big, nice, long lasting orgasm. And often. And that is gone. Just like that, gone together with the uterus.”* (pt 9, 59 years, leiomyosarcoma). Sexual intercourse also was less pleasant because of less sensitivity, more pain, and vaginal dryness. *“Because it's quite dry and it's sore, I tried quite a bit of lubricants but that gets sore, so that does affect my libido.”* (pt 13, 56 years, endometrial stromal sarcoma).

HCPs reported that treatment can lead to infertility, which can be related to all treatment modalities: “*especially pelvic surgery and radiation as well, and a very toxic chemotherapy, there is issues with fertility*.” (HCP 6, 41 years, surgical oncologist). Furthermore, female patients abruptly entered menopause due to removal of the ovaries or hormonal therapy for hormone receptor positive uterine sarcomas, and experienced complaints as hot flushes, night sweats, vaginal dryness, joint pain, and insomnia. Menopausal women were advised to stop taking hormone replacement therapy (HRT), leading to more symptoms: “*No, I was on HRT beforehand, and so, that was helping me with the hot flushes and everything, and then I had to stop taking it because of the cancer and everything. Then, the hot flushes came back..”* (pt 12, 54 years, leiomyosarcoma).

Patients and HCPs reported neurological symptoms such as numbness or tingling in the legs and/or feet, which could have an impact on mobility such as when riding a bicycle: *“Yes, I have a tingling feeling, when I am on the bicycle.”* (pt 9, 59 years, leiomyosarcoma). In some cases, patients were also limited in performing activities of daily living, for example in getting dressed *“I need help. I can shower myself independently, but then my feet aren’t washed. And with dressing too.”* (pt 9, 59 years, leiomyosarcoma) or performing household chores. Both patients and HCPs mentioned impairments in mobility, regarding driving a car, difficulties walking long distances, or walking stairs. Patients also reported lack of physical fitness and inability to do sports or leisure activities *“And on the other hand, in the past 1.5 years, I have been very much occupied with the fact that I can’t to run anymore, I can’t jump anymore. I was a gymnast. Yes, and I was always dancing. I still dance, but limited.”* (pt 9, 59 years, leiomyosarcoma).

All patients experienced varying degrees of fatigue and lacking energy during and sometimes also still after treatment:*”And now it is going alright, but I am just really tired.”* (pt 10, 55 years, adenosarcoma). One patient attributed fatigue to her hormonal changes “*The other thing is I do feel tired, exhausted because of the oestrogen effect, I'm sure.”* (pt 13, 56 years, endometrial stromal sarcoma). Additionally, patients had trouble sleeping, but the causes differed, such as having to go to the bathroom frequently *but now the tumour is pushing on my bladder, I'm up in the night all the time going to the loo”* (pt 12, 54 years, leiomyosarcoma) or because of worrying “*If I go to bed on time, at 10 PM, I don’t sleep and I am afraid of death. So I sleep badly. I go to bed when I feel tired.”* (pt 9, 59 years, leiomyosarcoma).

Some patients complained of shortness of breath or respiratory symptoms that only occurred during exertion: “*The most intense thing I feel is maybe on exertion the pressure on my chest.[..] So I swam one kilometer the other day. And then I do feel the pressure on my chest and what not.”* (pt 11, 54 years, leiomyosarcoma).

Pain also occurred in various circumstances and locations. Women had pain caused by the tumour itself: *“I've got a big lump in my stomach, and occasionally that’s a bit sore.”* (pt 12, 54 years, leiomyosarcoma)*,* but also back pain related to bone marrow stimulation during chemotherapy. Patients had postoperative pain, needed pain medication, and had to deal with its side-effects as well: *“had constipation and can’t no longer manage it through home remedies as used to before.”* (pt 4, 58 years, rhabdomyosarcoma).

HCPs described that extensive surgery for uterine STS could have both negative and positive consequences. Negatively, patients might need a colostomy, urostomy, or nephrostomy, or they can suffer from (abdominal wall) herniation: *“hernias or incisional issues long term because of the size and extent of the incisions”* (HCP 6, 41 years, surgical oncologist) or lymphedema. Positively, patients could experience a relief of symptoms, caused by the local effects of a large uterine tumour: *“The quality of life is improved enormously after the surgery. Because people get really sick from that sometimes, from those large tumours. Just literally get sick from it. And after the surgery they feel fitter and better and so on.”* (HCP 16, 42 years, surgical oncologist).

Other treatment-specific issues reported by patients with uterine STS included feeling ill or having flu-like symptoms, lower resistance to infections, hair and nail problems, eye and mouth problems, and skin changes during chemotherapy treatment. After radiation therapy for uterine sarcoma, women had a burning, painful skin, or blisters. Another patient had developed an ulcer near the anus.

Finally, women with metastatic tumours reported problems related to metastases or local treatment for sarcoma, such as difficulty eating and speaking, and removal of teeth because of metastasis in the jaw. Another patient mentioned being more sensitive to smell after lung surgery and weight gain as exercise was limited after treatment for a pelvic bone metastasis.

#### Mental health

Many patients experienced the diagnosis of cancer and moreover, a rare cancer, as a shock. They did not expect a sarcoma diagnosis because of a lack of symptoms or supposed benign cause of symptoms: “*And as [the baby] was born, they found what they thought was just like a little nodule in my womb, which they said is probably nothing. Everyone has these, it’s kind of like endometriosis or something similar to that, nothing to worry about. Yada yada yada. And then we went through further research on it, it actually turned out it was sarcoma. It came completely out of the blue because at no point had I any kind of symptoms and actually the surgeon even said, unfortunately this type of sarcoma is often symptomless. If we wouldn’t have found it because of this, you would probably have found it two weeks later and there would be nothing I could do.”* (pt 7, 39 years, endometrial stromal sarcoma). HCPs acknowledged this phenomenon as well and also emphasized that earlier misdiagnosis can have long-term effects on patients’ mental well-being: *“She has really struggled from a psychological point of view, partly because of earlier misdiagnosis, and she had that sort of protracted time to diagnosis and treatment. That’s brought with her a lot of anxiety, she’s had a change in career. She’s had a lot of psychological burden that she’s carried with her.”* (HCP 21, 33 years, physiotherapist).

Patients with uterine STS described feeling distressed because of the lack of knowledge about sarcoma, either because they did not understand the disease themselves *“And I think I still don’t quite understand what the difference between sarcoma and so-called normal cancer is. Also, specifically to sarcoma, because the other thing that you hear a lot from the doctors, it’s not your typical cancer, so what is typical cancer and how.."* (pt 7, 39 years, endometrial stromal sarcoma) or because there is less information about this rare type of cancer available: *“Because the population is so small, it’s just understanding how big are the risks if you do this or you don’t do that? Because there’s just not a lot of data, there’s not a lot of knowledge, it’s hard to understand myself, but it’s also hard to almost talk about it, because you’re not quite sure.”* (pt 7, 39 years, endometrial stromal sarcoma). One patient mentioned she felt the need to have contact with fellow patients to better understand how she was doing herself: *To have some sort of comparison: what’s the deal? What does that do to you? To compare a bit where you stand as well, or something. [..] somewhere you feel the need to sort of.. check”.* (pt 11, 54 years, leiomyosarcoma).

Being diagnosed with uterine STS also caused uncertainty regarding treatment, related to the morbidity of surgery or effectiveness of treatment: “*Yes. Well yes, yes. To the extent that it is effective, you also don’t know how long it will take before it comes back”* (pt 8, 59 years, leiomyosarcoma). Furthermore, patients with uterine sarcoma didn’t know what to expect in the future: *“And obviously, you know I’m only 39, I think just understanding what that means for your future life. I think that’s been the biggest blow for me and also because it’s not… It’s just so hard to digest but it’s also hard to know what questions to ask.”* (pt 7, 39 years, endometrial stromal sarcoma).

Women frequently experienced fear of recurrence, tainting the enjoyment of things in life: “*But every day I am afraid that I am going to start feeling something related to cancer again. So there is always dust over the beautiful things. And that is very big.”* (pt 9, 59 years, leiomyosarcoma) The anxiety often culminated at the moment of the next follow-up scan: *“Every time I go for a scan it is again the anxiety builds up until the diagnosis, I mean until the results come I am a bit anxious. That week is quite stressful going through the scans”* (pt 13, 56 years, endometrial stromal sarcoma). HCPs indicated that this is specific to patients with uterine sarcoma, because of the initial delay in diagnosis and/or unexpected news of suffering from a malignant disease. Patients also worried about the impact of their illness on loved ones. Some even decided not to tell family or friends about the severity of their situation: *“But in [country of origin] because they're far away we thought it best not to discuss and that's what I want. And I don't think it will make much difference. They would simply worry there.”* (pt 13, 56 years, endometrial stromal sarcoma).

Patients also reported being worried about having sex again, however they felt hesitant to discuss this with their medical team *“I have an intense fear of causing vaginal bleeding again, and am embarrassed to discuss the issue with my physician.”* (pt 4, 58 years, rhabdomyosarcoma). HCPs underlined the latter issue and recognized that concerns related to sexual issues are rarely brought up by patients themselves during appointments.

HCPs mentioned that women of reproductive age experience distress because they are faced with challenging decisions about having children as there is a high risk that treatment would cause infertility: *“And then there was before the radiation there was this issue with fertility if there should be an operation, I think an colorectal operation to change her ovary location or not. And she already had a child but then there was the issue if you want to have another and she had to discuss the pros and cons with her husband and her family.”* (HCP 12, 40 years, psychologist). According to HCPs, some patients experienced having to undergo fertility preservation as an additional burden: “*in women [fertility preservation] can delay [treatment] quite a bit. And that actually is a psychological burden, because ‘you say that I have a good prognosis, I want to have children, but I also want to be treated for this’.”* (HCP 10, 56 years, radiation oncologist).

Treatment for sarcoma also had an impact on the patients’ body image: women with uterine STS reported feeling less attractive compared with before being diagnosed*.* HCPs recognized this issue in their patients as well and reported this has great impact on patients’ self-esteem: “*I think, probably, having a hysterectomy and if there were other surgery, can affect their perception of themselves or the feeling that something is missing. So, that can also have effects on the psyche,” *(HCP 18, 49 years, medical oncologist).

Patients with uterine STS reported feeling more emotional or sad since diagnosis. Furthermore, they felt constantly reminded about their (previous) illness, either because of physical impairments or the psychological impact*: “for the psychological aspect I still get post traumatic. [..] Yes, probably women come with heavy periods and [as their GP]I'm sending them for operation it just gets me the flashback of what I've gone through. Especially with women who are menopausal.”* (pt 13, 56 years, endometrial stromal sarcoma).

#### Social health

Patients with uterine STS mentioned that their illness has had massive implications in their life and the activities they did or relations they had were significantly affected. For many patients, the effects of treatment caused limitations in participating in activities outside the house: “*I would maybe think twice about meeting someone of an evening if that was the day my eyes seemed to be bright-red and pouring, just because it’s not very pleasant.”* (pt 6, 57 years, leiomyosarcoma). This problem was also affirmed by HCPs. Side-effects of chemotherapy and radiation therapy also hampered social activities with others *“During chemotherapy and because of treatment side effects I chose to lower my engagement with others, diarrhea and nausea were the main causes.”* (pt 4, 58 years, rhabdomyosarcoma). Patients also had to give up leisure activities, hobbies, or activities with their (grand) children *“Swimming is not possible because of infections, this is important because this is "quality time" with my daughter.”* (pt 2, age missing, leiomyosarcoma).

The diagnosis also changed relationships with friends or family, due to a lack of understanding *“I've got a few close friends, but I've got one close friend who initially was really upset, and I wasn’t. I felt I was helping him more, and I was like, yes, you’ve just got to accept it.”* (pt 12, 54 years, leiomyosarcoma) or how people react to hearing about their illness. In some cases, they even lost contact with friends and women with uterine sarcoma felt isolated from others.

Patients with uterine STS also had to deal with loss of independence, either regarding housing *“I'm a bit worried about that, how I'm going to end up, or whether I should live with my family, or I might just save to move away. Luckily, I've got those options. I can do that financially, but I worry about whether I need to be near my family forever now, if you see what I mean.”* (pt 12, 54 years, leiomyosarcoma) or mobility *“I still can’t drive a car at this time. So the mobility, going somewhere, the freedom of going somewhere, the autonomy, the independence, is taken away.”* (pt 9, 59 years, leiomyosarcoma).

Patients often experienced limitations in continuing their studies or their original work, and often had to interrupt or adjust their working activities: *“I reduced the workload, I used to do a lot of out of hours and all, I've reduced that”* (pt 13, 56 years, endometrial stromal sarcoma). They felt it gave them the opportunity to focus on their recovery and to relax more. Others were forced to stop working entirely or chose to stop working. One patient experienced financial difficulties due to medical costs: *“I have a lot of financial problems due to the disease, I have far less money, only 250 euros for living.”* (pt 1, 46 years, sarcoma NOS).

The majority of HRQoL issues identified from the interview data are covered by the EORTC QLQ-C30 and EN-24 (see Table 2). Issues within the physical domain are assessed by both questionnaires. Heaviness or swelling of the abdomen, gynaecological symptoms, hormonal problems, treatment-related effects, weight problems, local effects of the tumour and infertility were missing from the questionnaires. The EORTC QLQ-C30 only assesses if patients are being worried, not specifying what causes them to worry. Changes in body image are assessed by the EORTC QLQ-EN24. The other mental health issues found in the interviews are not covered by either questionnaire. Nearly all social health issues are assessed by the EORTC QLQ-C30, although not always in detail (e.g. related to relationships with others). Changes in work or study capacity and the impact of a changed life are not included.

### Questionnaire review

All patients with uterine sarcoma reviewed the EORTC QLQ-C30 (Table 3) and EORTC QLQ-EN24, for topic and item relevance (Table 4). Based on the mean and prevalence scores, 3 of 5 items of the physical functioning scale, 1 item of 4 items of the emotional functioning scale, 1 of the 2 items of the symptom scale ‘pain’, the 2 items of the ‘nausea/vomiting’ scale, and 2 single items of diarrhoea and financial difficulties of the EORTC QLQ-C30 were less relevant.

**Table 3:** Assessment of EORTC QLQ-C30

No	Issue	Mean score (SD)	Prevalence ratio (%)	Remarks
1	Trouble doing strenuous activities	2.15 (0.99)	9 (69.2)	*“that is simply just walking uphill or if I'm carrying heavy shopping bags then I will get a little bit out of breath, which I didn’t used to” (pt 12, 54 years, leiomyosarcoma)* *“It’s not important to me whether or not I can carry that suitcase, but what is important is whether you have a partner who then carries your suitcase. Because I’d like to travel. So I’ve become completely dependent on somebody close to me who wants to travel with me. And wants to carry my things.”(pt 9, 59 years, leiomyosarcoma)*
2	Trouble taking a long walk	2.00 (0.82)	10 (76.9)	*“Do you have any trouble taking a long walk? Now, I don’t know what is considered long. I wasn’t sure, if I walk for an hour, that would probably be really tiring.” (pt 7, 39 years, endometrial stromal sarcoma)* *“…Then you’ll get the concept: what is a long walk? And if I find 40 km a long walk, but someone else finds 5 km a long walk, then you already have the wrong picture for such a questionnaire. Because then I’ll fill in: yes, I am limited. But then I’m talking about a whole different walk than someone else.” (pt 8, 54 years, leiomyosarcoma)*
3	Trouble taking a short walk	1.31 (0.63)*	3 (23.1)*	
4	Had to stay in bed or chair	1.46 (0.97)*	3 (23.1)*	
5	Needed help	1.23 (0.60)*	2 (15.4)*	
6	Limited in work or daily activities	1.62 (0.77)	6 (46.2)	*“I could go to work, and I could do my job at the moment, but I choose not to, because…So it’s not debilitating me so much that I can’t work, but I’m making a kind of conscious decision to not go to work, because I think it’s more important to look after my mental wellbeing and stay home. So I could do my job, but I think it’s better to take time out.”(pt 6, 57 years, leiomyosarcoma)*
7	Limited in hobbies or leisure time activities	2.15 (1.21)	8 (61.5)	
8	Shortness of breath	1.77 (0.73)	8 (61.5)	
9	Pain	1.62 (0.96)	5 (38.5)	*“But my bones started to bother me very much, so the chemo does have that effect.” (pt 8, 54 years, leiomyosarcoma)*
10	Need to rest	2.23 (1.01)	10 (76.9)	
11	Trouble sleeping	2.38 (1.04)	10 (76.9)	*“ but a lot of it is simply the tumour pressing on my bladder, I think, where I have to get up in the night.” (pt 12, 54 years, leiomyosarcoma)* *“This is difficult at time. After the surgeries. Then you get sleeping pills.” (pt 9, 59 years, leiomyosarcoma)*
12	Felt weak	2.15 (0.90)	10 (76.9)	
13	Lack of appetite	1.69 (1.11)	5 (38.5)	
14	Nausea	1.46 (0.88)*	4 (30.8)	
15	Vomiting (n = 12)	1.25 (0.87)*	1 (9.1)*	
16	Constipation	1.92 (1.12)	6 (46.2)	
17	Diarrhoea	1.46 (0.78)*	3 (23.1)*	*“I suffer from constipation. I take those sachets [laxatives] for that, and then after three days I suffer from diarrhoea. So all that also is very annoying.” (pt 8, 54 years, leiomyosarcoma)*
18	Tiredness	2.38 (1.04)	10 (76.9)	
19	Pain interfering with daily activities	1.31 (0.86)*	1 (7.7)*	
20	Difficulty concentrating	2.08 (1.04)	7 (53.8)	
21	Felt tense	1.46 (0.78)*	4 (30.8)	
22	Felt worried	2.23 (0.93)	10 (76.9)	*“because I worried a little bit about the future, and how I'm going to plan things.”(pt 12, 54 years, leiomyosarcoma)*
23	Felt irritable	1.69 (0.86)	6 (46.2)	
24	Felt depressed	1.62 (0.96)	5 (38.5)	*“Depressed? I would say sometimes it’s more sad. Depressed, I would say not at all, but it’s just again me just feeling a bit sad, so I’ll say not at all.” (pt 12, 54 years, leiomyosarcoma)*
25	Difficulty remembering	1.85 (1.14)	6 (46.2)	*“Sometimes I have problems to find the right words, it is like Alzheimers disease” (pt 2, age missing, leiomyosarcoma)* *“Yes, I sometimes have the idea that I have dementia, can’t think of things or..[..] many things pass me by yes. And I just can’t think of things.” (pt 10, 55 years, adenosarcoma)*
26	Interference with family life	2.08 (1.19)	6 (46.2)	
27	Interference with social life	2.08 (1.12)	7 (53.8)	
28	Financial difficulties	1.62 (1.04)	3 (23.1)*	*“Look, if you have a permanent job and you’re in this, then your salary will continue to be paid. So that part, financially, that’s just less income, so to speak. So you notice that of course. But it doesn’t mean that we’re getting into trouble because of that.” (pt 10, 55 years, adenosarcoma)* *“You go from 100 percent salary to 70 percent. [..] We have also bought another house. It had to be completely redesigned. The bathroom. Everything on the ground floor, because at first I thought I would get in a wheelchair. Everything downstairs. [..] I did not get into a difficult situation. But it's looming. There have been a lot of expenses.” (pt 8, 59 years, leiomyosarcoma)*
29	Overall health (n = 12)	5.33 (1.23)	11 (84.6)	*“I've just put nothing major, sore throat, slight aches in stomach, upper body, slightly fluey, so I've put number four” (pt 12, 54 years, leiomyosarcoma)* *“Well, because then you just really feel the effect of the chemo. So the tiredness. That the food really less… So nauseous. And the food tastes less. And you’re busy trying to get back on track. So that health, there you really feel the effect during the chemo of course, yes, from that chemo.”(pt 11, 54 years, leiomyosarcoma)* *“So when you’re asking about my health, like compared to before I was ill, how has my health changed, or compared to when I was feeling my worst, how has my health changed. So it’s kind of a bit of a nebulous question, in a way, if that makes sense.” (pt 6, 57 years, leiomyosarcoma)*
30	Overall quality of life (n = 12)	5.17 (1.27)	11 (84.6)	*“Yesterday evening we sat together with everyone outside again with the family and that’s it.. Those are the things that make you feel better.[..] But as nice as it is, you’re going to bed on time. Because you can’t take it.[..] Yes, but if I keep sitting and it gets 11 o’clock then I have to again the next day… Then I’ll be even more tired the next day.”(pt 10, 55 years, adenosarcoma)*

*Relevance score below cut-off value

*Missing issues*

Muscle and limb pain

Loss of independence

What symptom or side-effect is the most limitative

Worries about the future

Worries about impact on loved ones

How mental health in linked in with physical health

Need for support (psychological support or practical support)

In terms of the EORTC QLQ-EN24, both items of the lymphedema symptom scale, 1 of 4 items of the urological symtom scale, 2 of 5 items of the gastrointestinal symptom scale, and the single item ‘tingling or numbness in hands or feet’ were less relevant based on the mean and prevalence scores. Of note, the items of sexual/vaginal problems and sexual enjoyment scale were completed by 7 out of 13 of uterine sarcoma patients.

During the qualitative assessment of the EORTC QLQ-C30 and the EORTC QLQ-EN24, patients and HCPs reported whether some items were missing, or could be changed or deleted. Their recommendations for changes and missing issues are included in Tables 3 and 4. No suggestions were made for deletion of items.

**Table 4:** Assessment of EORTC QLQ-EN24

31	Swelling legs	1.31 (0.75)*	2 (15.4%)*	
32	Heaviness legs	1.23 (0.60)*	2 (15.4%)*	
33	Pain in lower back and/ or pelvis	1.54 (0.88)	5 (38.5%)	*“it was kind of very strange low backpain, which I diagnosed as being dehydrated and my kidneys complaining. But when I spoke to Dr [..], she said it was probably the bone marrow injection that I’d given myself that had stimulated my bone marrow and caused some bone pain.” (pt 6, 57 years, leiomyosarcoma)*
34	Urge to pass urine, hurry to get to the toilet	2.15 (1.14)	8 (61.5%)	*“Urge to pass urine, yes, every night.” (pt 12, 54 years, leiomyosarcoma)*
35	Passed urine frequently	2.15 (0.90)	10 (76.9%)	
36	Leaking of urine	1.54 (0.78)	5 (38.5%)	*“loss of urine not always but when I need to cough” (pt 2, age missing, leiomyosarcoma)*
37	Pain or burning feeling when passing urine	1.38 (0.65)*	4 (30.8%)	
38	Urge to move bowels, hurry to get to the toilet	1.54 (0.88)	5 (38.5%)	
39	Leakage of stools	1.00 (0)*	0 (0%)*	
40	Troubled by passing wind	1.54 (0.78)	5 (38.5%)	*“flatulence and therefore stomach pain” (pt 2, age missing, leiomyosarcoma)*
41	Cramps in abdomen	1.38 (0.77)*	3 (23.1%)*	*“When you say cramps, do you mean like when you go to the loo, or sort of period pain or that sort of thing? So, it’s not just aching? [..] because I didn’t know whether that meant period or diarrhoea cramps, or wind even that you might have. I wasn’t too clear what that meant.”(pt 12, 54 years, leiomyosarcoma)*
42	Bloated feeling in abdomen	1.58 (0.79)	5 (41.7%)	*“I do get bloated after I've eaten, but I know I've been told to have small, regular meals, but occasionally, if I have a slightly bigger meal which maybe I shouldn’t be doing anyway, I do get bloated, but that doesn’t last a long time “(pt 12, 54 years, leiomyosarcoma)* *“So you’re already not inclined to eat so much that you’re full, because then you’ll get such a… You also don’t know how your body should do it, so then you don’t know at all how to do it anymore. So you’re already not inclined to eat until you’re so full that you think like, okay I’m full. Because then it is too heavy.”(pt 10, 55 years, adenosarcoma)*
43	Tingling or numbness in hands or feet	1.23 (0.44)*	3 (23.1%)*	
44	Aches or pains in muscles or joints	1.77 (0.60)	9 (69.2%)	*”I get in the upper body [..] and that lasts for three or four days, and mainly around the time I start with injections. So, it starts about three days after the treatment.” (pt 12, 54 years, leiomyosarcoma)*
45	Hair loss	2.00 (1.41)	5 (38.5%)	*“I was originally on doxorubicin; I could literally pull my hair out and it would… I would just hold it and it would come out in chunks.”(pt 12, 54 years, leiomyosarcoma)*
46	Different taste of food and drink	1.77 (1.17)	5 (38.5%)	*“Yes, it all tastes like bread or something. Yes, it feels just like a… It’s also different in your mouth. It really feels like a ball of flour instead of.. You could just as well eat porridge, because I just can’t swallow it. It just takes effort. Your taste is less.”(pt 8, 54 years, leiomyosarcoma)*
47	Felt physically less attractive	2.31 (1.10)	9 (69.2%)	*“It’s that my stomach sticks out with the tumour and I'm quite aware of that and I feel a bit self-conscious and try to wear clothes that cover it up, and when it’s not… I'm sure no one does notice, but I just feel unattractive because of that. And obviously, because I'm going a bit bald now.” (pt 12, 54 years, leiomyosarcoma)*
48	Felt less feminine	1.92 (1.12)	7 (53.8%)	*“Yes. If you don’t have hair anymore, then you actually are less feminine I have to say.” (pt 8, 54 years, leiomyosarcoma)* *“And I do still have my eyebrows and eyelashes. I would say if I lost those, the answer to that question would be different,” (pt 12, 54 years, leiomyosarcoma)*
49	Interest in sex	1.69 (0.75)	7 (53.8%)	
50	Extent sexually active	1.69 (0.86)	7 (53.8%) 15	*“I think that’s because I’m worried it’s going to make me start bleeding again “-(pt 6, 57 years, leiomyosarcoma)* *“emphasize fear of having sexual intercourse”(pt 4,58 years, rhabdomyosarcoma)*
51	Vagina felt dry (n = 7)	3.00 (1.29)	6 (85.7%)	
52	Vagina felt short/tight (n = 7)	2.00 (1.00)	4 (57.1%)	
53	Pain during sexual activity (n = 7)	2.14 (1.22)	4 (57.1%)	
54	Sexual activity was enjoyable (n = 7)	2.29 (1.11)	5 (71.4%)	

*Relevance score below cut-off value

*Missing issues*

Psychological overlay of sexual problems

Menopausal symptoms (hot flushes, weight gain)

Vaginal bleeding

Rectal bleeding

Postoperative fistulas

## Discussion

This is the first study to investigate HRQoL issues, both from the perspective of patients and of HCPs, and how they can be measured in uterine STS patients using a mixed-method design. The most commonly reported physical health issues were related to sexual and functional problems, urological symptoms, fatigue, pain, and treatment-related adverse effects. Hormone-related issues and a variety of gastrointestinal symptoms were also found. Regarding mental health, fears (about having sex, of recurrence, or of death), a changed body-image, and dealing with a lack of knowledge of sarcoma had a strong impact on HRQoL. Social health issues were related to changes in relationships with others, limitations in undertaking activities, loss of independence, changes in work or study capacity and financial difficulties.

Physical health issues reported by uterine sarcoma patients in our study overlap with issues in other gynaecological cancers. Similar to other female cancers [21], women with uterine sarcoma entered menopause as a result of treatment or, in the case of hormone sensitive tumours, were prohibited from using hormone replacement therapy. This led to many hormone-related symptoms, also known as the genitourinary syndrome of menopause (GSM) [22]. In addition to GSM, limited sexual desire because of chemotherapy-related toxicity such as nausea, fatigue, and impacted body image due to chemotherapy-induced alopecia (i.e. feeling less attractive or feminine) are known to contribute to sexual dysfunction in female cancer patients [21]. Our study found the same issues and impact on sexual functioning. The presence of gastrointestinal and urological symptoms also matches with findings in previous studies in uterine sarcoma patients, as well as in cervical and endometrial cancer survivors [15, 23]. However, in the latter group pelvic radiation is known to contribute to symptom severity. As this treatment modality is not administered routinely for uterine STS, it is not clear if the severity is comparable as well.

The HRQoL issues in the psychosocial domain are similar between patients with uterine STS and other gynaecological cancers. Patients recalled the sarcoma diagnosis coming as a shock. A survey conducted among gynaecological sarcoma patients by the British sarcoma charity showed a similar pattern in the diagnostic trajectory, with 53% of respondents going into surgery without suspecting their problem might be cancerous [16]. HCPs thought diagnostic delay and subsequent shock of diagnosis led to an increased fear of recurrence. No studies investigated impact of diagnostic trajectory on fear of recurrence specifically, but diagnostic delay could cause psychological distress [24]. Patients also reported fears of having sex, a domain of sexual dysfunction in female cancer patients that has been poorly reported in literature. However, there is some evidence of psychoeducational interventions that can reduce sexual fears [25]. Uterine STS patients struggled with the lack of knowledge about their disease. This was also found in 32% of a heterogeneous sarcoma survivor population saying they found it difficult having to explain their disease [26]. Additionally, comparable results were found regarding the desire to have contact with fellow sarcoma patients [26].

A variety of cancer-generic HRQoL issues were reported as well, such as fatigue, pain, chemotherapy- and radiotherapy-related toxicities, feeling tense or anxious, and impact on work, finances, and social life. These issues have been well described in the cancer population in general and are mostly covered by the EORTC QLQ-C30 questionnaire [13].

For the majority of the items of the EORTC QLQ-EN24, uterine sarcoma patients gave relevance scores higher than the cut-off values. The cut-off value for relevance was lower than the example (i.e. mean > 2.0) given in the EORTC QLG module development guidelines to be more inclusive for issues in the current explorative analysis [20], but needed to be higher than 1.0 to indicate any degree of relevance. The module development guidelines are more focused on specific tumour sites in homogeneous and common cancers, whereas the current study aims to search for a measurement strategy in a rare and heterogeneous population using existing items from item banks or libraries [27]. Guidelines for the use of customized item lists from the EORTC QLG portfolio are currently in development [28], and data-driven cut-off points have yet to be defined for this process. We feel that, despite the current small number of patients, some issues can be declared as ‘insufficiently relevant’ as low relevance scores for some items can be explained by differences in phenotype or treatment between uterine STS and carcinoma patients. Lymphedema is more common in endometrial cancer patients, as they have to undergo pelvic lymph node dissection [29] and are more frequently overweight [30]. Urological and gastrointestinal symptoms are mostly related to pelvic radiation, which is not part of standard first line treatment for uterine sarcoma, but can occur particularly with locoregional relapse. Tingling/numbness of the legs and feet can be caused by treatment with docetaxel, but is given less commonly than paclitaxel/carboplatin in endometrial cancer.

This study provides insight in HRQoL issues experienced by uterine STS patients, following the HRQoL conceptual model that is used in oncological clinical trials and health care [10, 31]. Although many issues match with issues reported in other urogenital cancers, it is valuable to verify these issues in the context of sarcoma diagnosis and treatment and include them in HRQoL assessment in future research. Furthermore, some issues appearing to be specific for sarcoma were identified, such as the impact of unexpected diagnosis and lack of knowledge about the disease. With regard to measurement of HRQoL, the EORTC QLQ-C30 seems to be an adequate instrument to assess cancer-generic issues in the uterine STS population. Items that had low relevance scores (e.g. nausea/vomiting, diarrhoea) are known to be less relevant in a ‘cancer-survivor’-population [32]. A survivor-specific module is currently in development [32], and may be used in sarcoma patients as well. The EORTC QLQ-EN24 is also applicable in uterine STS patients, although some items seem to be redundant based on relevance scores. On the other hand, some issues specific to the sarcoma population could be added such as menopausal symptoms, pressure or heaviness in the abdomen, or the lack of contact with fellow patients. Items about sexuality are deemed relevant, looking at both relevance scores and interview analysis, however were not completed by all patients. This phenomenon was previously seen in validation studies for the EORTC QLQ-EN24 as well [14, 33]. Given the fact that HCPs acknowledged during the interviews that sexual problems are discussed infrequently and that insufficient psychosexual support is offered, it appears to be important to include items about sexuality in HRQoL assessment in uterine STS patients.

Although this is the first study to investigate HRQoL in this patient group specifically, some limitations need to be taken into account. First, the sample size of uterine sarcoma patients is small. Furthermore, no gynaecologists were interviewed despite their involvement in some countries in follow-up care for women treated for localized uterine STS. This is caused by the fact that the larger mixed method study, in which the current data was collected, was aimed at the heterogeneous sarcoma population and HCPs working in sarcoma care [17]. Systematic review of the literature revealed that limited data was available on HRQoL in uterine sarcoma patients, prompting us to conduct analysis on data available for this subgroup. Additional issues could have come up if more uterine sarcoma patients and gynaecologists were included. Second, several interviews were conducted during the global COVID-19 pandemic which may have influenced HRQoL of uterine sarcoma patients. However, patients were explicitly asked about their sarcoma-specific experience and we found no mention of the COVID-19 pandemic or its restrictions in the transcripts. Third, we had no access to the qualitative data on which items of the EORTC QLQ-C30 and EORTC QLQ-EN24 were originally based, so we could not distinguish if they reflect the issues found in our interviews or need to be adjusted for the uterine STS population based on the comments in our interviews. Correlations between specific issues and overall health or quality of life could be analysed using item scores instead of relevance scores that were collected in our current study. Finally, we did not assess comprehensibility (i.e. whether the items are understood by patients as intended) of the EORTC QLQ-C30 and EORTC QLQ-EN24, which is a third aspect of content validity [34]. Comprehensibility of these questionnaires has already been assessed in their validation-studies [13, 14] and we assumed that it would be similar in the context of uterine sarcoma patients.

The aforementioned insights in specific HRQoL issues experienced by uterine sarcoma patients will help create awareness in HCPs caring for patients with uterine sarcomas, improve provision of information to patients, and facilitate personalized supportive care. Our findings also offered guidance for the development of a flexible measurement strategy for HRQoL assessment in sarcoma patients, using existing cancer-generic and location-specific questionnaires combined with tailored item lists. The next phase of this mixed-method study is currently ongoing [17], in which the results reported here are incorporated. HRQoL issues based on interviews will be reviewed by a larger number of sarcoma patients with the aim to determine which HRQoL issues are sufficiently relevant to be considered for sarcoma subgroups, including uterine STS. The new measurement strategy will facilitate collection of sarcoma-specific patient-reported outcome data in clinical trials and observational studies, and help determine patient groups for whom care can be personalized.

## Conclusion

Patients with uterine STS experience a variety of issues in the physical, mental, and social domain that impact their HRQoL. Some issues were specific for the tumour location, others were related to the rare sarcoma diagnosis specifically. The majority of items in the existing EORTC QLQ-C30 and EORTC QLQ-EN24 questionnaires were relevant for HRQoL, although some additional issues were missing. For future research, a flexible measurement strategy combining cancer-generic, location- and sarcoma-specific items is recommended to assess HRQoL in uterine sarcomas.

## Supplementary Information

**Additional file 1.** Interview guides.

**Additional file 2.** COREQ checklist.

**Additional file 3.** Quotes from interviews.

## Data Availability

The datasets generated and/or analysed during the current study are not publicly available due them containing information that could compromise research participant consent but are available from the corresponding author on reasonable request.

## Abbreviations

EORTC QLQ-C30: EORTC quality of life core questionnaire 30
EORTC QLQ-EN24: EORTC QLQ-endometrial cancer module
GSM: Genitourinary syndrome of menopause
HCP: Health care professionals
HRQoL: Health-related quality of life
HRT: Hormone replacement therapy
STS: Soft-tissue sarcoma

## Footnotes

1 *indicates patient or HCP ID, age at study participation, sarcoma histological subtype for patients or specialism for HCPs.*
