# Supplementary material for: ‘I thought I had fibroids, and now I don’t’: a mixed method study on health-related quality of life in uterine sarcoma patients
Source: Health Qual Life Outcomes. 2022 Apr 20;20:65. doi: 10.1186/s12955-022-01971-5 (PMC9020416; doi:10.1186/s12955-022-01971-5)
Supplement: Supplementary file 1 — Additional file 1. Interview guides. [file 12955_2022_1971_MOESM1_ESM.docx]

Additional file 1: interview guides

**Phase 1A - Patient Interview Guide:**

**Outline**

- Introduce the study and the reasons we are asking them to participate
- Assure them about maintaining confidentiality
- Explain the need to gather some personal information about them for the research study
- Ask them first about current issues/ experiences they are having because of having been diagnosed with sarcoma
- Explain what we expect them to rate today *(i.e. the EORTC QLQ-C30 and if relevant site specific module)*
- Present EORTC QLQ-C30 and if relevant the site specific module and ask them to rate it *(explain this is a cancer QOL questionnaire for patients under treatment)*
- Ask them to tell about any issues they feel are missing from the questionnaires
- Thank them for sharing their experiences with us and providing feedback on our questionnaire

**PHASE 1:**

**GENERATION OF RELEVANT HEALTH-RELATED QUALITY OF LIFE ISSUES *(RED FONT INDICATES INTERVIEWER’S GUIDE****)*

The study coordinator should begin the interview with some introductory remarks to explain the nature and purpose of the research study. For example:

- Introduce the study and the reasons we are asking them to participate

***We are asking for your help in devising a questionnaire which will be used to monitor the experiences of people who have been diagnosed with sarcoma. I would like to ask you a few things about your health.***

- Assure them about maintaining confidentiality

***Remember all of this information you are providing will remain confidential and your data will be assigned a unique research number so your name will not appear anywhere these data are used in the future.***

- Explain the need to gather some personal information about them for the research study

***In order to provide some background information for our research I would like to gather some personal information about your life circumstances including current work and relationship status, and about your health (e.g. date of diagnosis; past and current treatment; time since last treatment).*** (Use an appropriate list to complete with the patient)

- Ask them first about their illness, but keep this brief

***Can you tell me about your illness/condition?***

- Ask them about current issues/ experiences they are having because of being diagnosed with sarcoma

***What are the most important things you experienced? Or can you think of any additional experiences?***

***Follow-up questions***

The use of follow-up questions or "probes" will be required in the majority of interviews. The appropriate wording is dependent on the topic at hand, but should always be in an open, non-judgmental way. For example:

· If the answer is too general and indefinite, the follow-up may be

***In what way? Just how do you mean? Can you give me an example?***

· If the answer is incomplete, the questions may be:

***Any other reasons? Would you tell me a little more about that?***

It may be useful to prompt the patient to consider specific domains, especially if the literature review has suggested that these may be relevant to the patient group. Some examples are shown:

**· *What are you not able to do that you would formerly do before your illness, any why?***

**· *Are you limited in normal daily activities (e.g.- work, household, shopping, taking care of the children, sports) compared to before your illness? What is it that limits you?***

**· *Are you undertaking fewer social activities (e.g.-hobbies, meeting up with friends) and why?***

**· *Have changes in relationships with family/friends occurred?***

**· *Do you have financial problems or worries due to your illness (e.g. insurance, mortgage, loans)?***

**· *Have your personal feelings changed (e.g.-satisfaction with life, spirituality)?***

**· *Has your emotional wellbeing changed (e.g.-feelings of anxiety or worrying)?***

**· *Are there any other issues or comments you would like to make regarding your illness and treatment and your quality of life?***

- Explain what we expect them to rate today *(i.e. the QLQ C-30 and the site specific module)*

***Today we are asking you to rate two questionnaires and provide us with your feedback about them.***

***The QLQ C-30 is an existing questionnaire used for patients under treatment for many different types of cancer. The … module is specifically designed for patients who are currently under treatment for….cancer.***

- Present QLQ-C30 and the site specific module and ask them to rate it *(explain this is a cancer QOL questionnaire and if relevant the site specific module for patients under treatment)*

***This is an existing questionnaire that asks about patients under treatment for cancer and their quality of life. These questions may also be of value for patients with sarcoma.***

***Today we would like you to rate these questions according to ‘Relevance’ .***

***“Relevance” refers to the frequency with which a specific issue occurs and, when it occurs, the trouble it may cause. Thus, the more frequently an issue occurs and the more trouble it causes, the more relevant it will be.***

***Could you please indicate for each question separately the extent to which you find it relevant for you?***

1. ***Not at all relevant***
2. ***A little relevant***
3. ***Quite relevant***
4. ***Very relevant***

**(Place the QLQ-C30 and the module before the patient who rates it)**

- Ask them to tell us about any issues they feel are missing from the questionnaires

***Now that you have completed both these questionnaires can you please tell whether you think these questionnaires describe sufficiently your current health? If no: please name the issues you think need to be added so it will be able to describe your functioning?***

***Can you tell me about any issues you feel are missing related to the experiences you may have had as a result of being diagnosed with sarcoma which you feel has affected your quality of life, in both a positive or negative way.***

***Follow-up questions***

·The interviewer may use additional neutral probes, e.g*.:*

**·*Can you tell me more about that? Can you think of additional experiences?***

· Other follow-ups could ask:

**·*What makes you think this? What was there in the question that made you feel that way?*"**

It may be useful to prompt the patient to consider specific domains, especially if the literature review has suggested that these may be relevant to the patient group. Some examples are shown:

**·** ***Do you have any other symptoms not mentioned in the questionnaire?***

**· *Do you have other problems with your physical functioning/health/changes in sleep patterns***

- Thank them for sharing their experiences with us and providing feedback on our questionnaire

***Thank you very much for your time today and for agreeing to participate in this research study. Your personal information and experience is very valuable to us in the development of this Sarcoma questionnaire.***

**Phase 1a- Health care professional Interview Guide:**

***Outline***

- The interviews with health care professionals complement the patient interviews. They may be

able to identify important but uncommon issues that may not be found in the relatively small

number of Phase 1 patient interviews.

- It is most useful to conduct the health professional interviews after generation of relatively complete lists of issues, that is after the literature review and after completion of (most of) the patient interviews.
- Introduce the study and the reasons we are asking them to participate
- Assure them about maintaining confidentiality
- Explain the need to gather some personal information about them for the research study
- Ask them first about issues/ experiences sarcoma patients face because of having been diagnosed with sarcoma
- Explain what we expect them to rate today *(i.e. the EORTC QLQ-C30 and if relevant site specific module)*
- Present EORTC QLQ-C30 and if relevant the site specific module and ask them to rate it *(explain this is a cancer QOL questionnaire for patients under treatment)*
- Ask them to tell about any issues they feel are missing from the questionnaires
- Thank them for sharing their experiences with us and providing feedback on our questionnaire

**PHASE 1a:**

**GENERATION OF RELEVANT HEALTH-RELATED QUALITY OF LIFE ISSUES *(RED FONT INDICATES INTERVIEWER’S GUIDE****)*

The study coordinator should begin the interview with some introductory remarks to explain the nature and purpose of the research study. For example:

- Introduce the study and the reasons we are asking them to participate

***We already have a questionnaire assessing quality of life aspects of cancer patients in general. Quality of life aspects relevant to specific diagnostic patient groups are not included in this questionnaire. We are asking your help in devising a questionnaire which will be used to assess the quality of life of patients who have sarcoma.***

- Assure them about maintaining confidentiality

***Remember all of this information you are providing will remain confidential and your data will be assigned a unique research number so your name will not appear anywhere these data are used in the future.***

- Explain the need to gather some personal information about them for the research study

***In order to provide some background information for our research I would like to gather some information about you and your working experience in sarcoma care (age, sex, your function in sarcoma care, years of working experience in health care and specifically in sarcoma care).***

- Ask them first about their experiences, but keep this brief

***Could you tell me what you see in your practice with sarcoma patients ?***

***Follow-up questions***

The use of follow-up questions or "probes" will be required in the majority of interviews. The appropriate wording is dependent on the topic at hand, but should always be in an open, non-judgmental way. For example:

· If the answer is too general and indefinite, the follow-up may be

***In what way? Just how do you mean? Can you give me an example?***

· If the answer is incomplete, the questions may be:

***Any other reasons? Would you tell me a little more about that?***

It may be useful to prompt the health care professional to consider specific domains, especially if the literature review has suggested that these may be relevant to the patient group. Some examples are shown:

- ***What are the most frequent reported symptoms that potentially diminish quality of life (during treatment/follow-up) of sarcoma patients?***
- ***Do you assess patients’ symptoms and quality of life (and how)?***
- ***What difficulties do you encounter when assessing patients’ symptoms and quality of life?***
- ***What are patients not able to do that they would formerly do before their illness, any why?***
- ***Are they limited in normal daily activities (e.g.- work, household, shopping, taking care of the children, sports) compared to before their illness? What is it that limits them?***
- ***Are they undertaking fewer social activities (e.g.-hobbies, meeting up with friends) and why?***
- ***Do changes in relationships with family/friends occur?***
- ***Do they have financial problems or worries due to their illness (e.g. insurance, mortgage, loans)?***
- ***Has their emotional wellbeing changed (e.g.-feelings of anxiety or worrying)?***
- ***Are there any other issues or comments you would like to make regarding sarcoma diagnosis and treatment and quality of life of sarcoma patients?***
- Explain what we expect them to rate today *(i.e. the QLQ C-30 and the site specific module)*

***Today we are also asking you to rate one/two questionnaires and provide us with your feedback about them. We would like you to rate the QLQ C-30, an existing questionnaire used for patients under treatment for many different types of cancer. The … module is specifically designed for patients who are currently under treatment for….cancer.***

- Present QLQ-C30 and the site specific module and ask them to rate it *(explain this is a cancer QOL questionnaire and if relevant the site specific module for patients under treatment)*

***This is an existing questionnaire that asks about patients under treatment for cancer and their quality of life. These questions may also be of value for patients with sarcoma.***

***Today we would like you to rate these questions according to ‘Relevance’ . “Relevance” refers to the frequency with which a specific issue occurs and, when it occurs, the trouble it may cause. Thus, the more frequently an issue occurs and the more trouble it causes, the more relevant it will be.***

***Could you please indicate for each question separately the extent to which you find it relevant for your patients?***

1. ***Not at all relevant***
2. ***A little relevant***
3. ***Quite relevant***
4. ***Very relevant***

**(Place the QLQ-C30 and the module before the HCP who rates it)**

- Ask them to tell us about any issues they feel are missing from the questionnaires

***Now that you have completed both these questionnaires can you please tell whether you think these questionnaires describe sufficiently the health status of the sarcoma patients? If no: please name the issues you think need to be added so it will be able to describe their functioning?***

***Can you tell me about any issues you feel are missing related to the experiences patients may have had as a result of being diagnosed with sarcoma which you feel has affected their quality of life, in both a positive or negative way.***

***Follow-up questions***

·The interviewer may use additional neutral probes, e.g*.:*

**·*Can you tell me more about that? Can you think of additional experiences?***

· Other follow-ups could ask:

**·*What makes you think this? What was there in the question that made you feel that way?*"**

It may be useful to prompt the HCP to consider specific domains, especially if the literature review has suggested that these may be relevant to the patient group. Some examples are shown:

**·** ***Do patients have any other symptoms not mentioned in the questionnaire?***

**· *Do patients have other problems with their physical functioning/health/changes in sleep patterns?***

Summarise the issues raised during the interview and identify which issues are the most important.

- ***We have discussed a number of things. These are…***
- ***Out of everything we have talked about today, what are the top three needs you see in practice and how could they be better met?***
- ***Is there anything else about needs related to cancer treatment or survivorship that you can think of that we have not already discussed?***
- Thank them for sharing their experiences with us and providing feedback on our questionnaire

***Thank you very much for your time today and for agreeing to participate in this research study. Your personal information and experience is very valuable to us in the development of this Sarcoma questionnaire***
