# Supplementary material for: ‘I thought I had fibroids, and now I don’t’: a mixed method study on health-related quality of life in uterine sarcoma patients
Source: Health Qual Life Outcomes. 2022 Apr 20;20:65. doi: 10.1186/s12955-022-01971-5 (PMC9020416; doi:10.1186/s12955-022-01971-5)
Supplement: Supplementary file 3 — Additional file 3. Quotes from interviews. [file 12955_2022_1971_MOESM3_ESM.docx]

Additional file 3: quotes from interviews

**Patient quotes**

| **PHYSICAL HEALTH** |
| --- |
| **1. Gastrointestinal symptoms** |
| 1.1 Bloated feeling of the abdomen |
| feeling bloated and uncomfortable due to the pressure on my abdomen (pt 3, 61 years, undifferentiated spindle cell sarcoma) |
| 1.2 Pressure in the abdomen |
| …felt a lot of pressure on my bladder (pt 3, 61 years, undifferentiated spindle cell sarcoma) |
| And on December 31st, I didn’t know how to sit. I had such a painful.. Yeah, like my intestines wanted to come out. (pt 10, 55 years, adenosarcoma) |
| 1.3 Feeling a mass in the abdomen |
| And for half a year, I actually felt it grow (pt 11, 54 years, leiomyosarcoma) |
| .. meanwhile my belly was growing enormously. I even said to a colleague: ‘It seems like I am four months pregnant.’ (pt 9, 59 years, leiomyosarcoma) |
| felt a lump lower down (pt 3, 61 years, undifferentiated spindle cell sarcoma) |
| I've got a big lump in my stomach (pt 12, 54 years, leiomyosarcoma) |
| 1.4.1 Constipation |
| The radiotherapy affected me negatively in the abdominal area. I suffered diarrhea and constipation and ulcer in the anus area. (pt 4, 58 years, rhabdomyosarcoma) |
| I have constipation. I take sachets [laxatives] for that, and then after three days I get diarrhea. So that is all very annoying as well. (pt 8, 59 years, leiomyosarcoma) |
| Need for oral supplement for bowel function (pt 5, 46 years, PEComa) |
| 1.4.2 Diarrhoea |
| The radiotherapy affected me negatively in the abdominal area. I suffered diarrhea and constipation and ulcer in the anus area. (pt 4, 58 years, rhabdomyosarcoma) |
| 1.4.3 Tingling sensation in hips when I need to open my bowels |
| I also experience a tingling sensation in the hips when I need to open my bowels. (pt 3, 61 years, undifferentiated spindle cell sarcoma) |
| 1.5 Lack of appetite |
| I have no appetite sometimes, it depends on the medication. (pt 2, age missing, leiomyosarcoma) |
| Before my operation [….] my appetite went down (pt 3, 61 years, undifferentiated spindle cell sarcoma) |
| Well yes, I don’t have much of an appetite. (pt 10, 55 years, adenosarcoma) |
| Yes, these are all phases. You’re nauseous the first couple of days. And then you don’t want to eat anyway. (pt 11, 54 years, leiomyosarcoma) |
| No, I do eat. But I can’t eat everything. (pt 8, 59 years, leiomyosarcoma) |
| 1.6 Gastro-esofageal reflux |
| Heartburn is very annoying. (pt 8, 59 years, leiomyosarcoma) |
| 1.7 Nausea/ Vomiting |
| Well, one day I woke up to, oh, I feel a little bit sick and I took an anti-sickness tablet (pt 12, 54 years, leiomyosarcoma) |
| During chemotherapy and because of treatment side effects I chose to lower my engagement with others, diarrhea and nausea were the main cause. (pt 4, 58 years, rhabdomyosarcoma) |
| But then I had to lay in bed for a week feeling sick, I was really just nauseous and I could only lay in bed. I was really sick. (pt 10, 55 years, adenosarcoma) |
| Yes, so then you have this nausea. Then you get this medication to suppress the nausea. (pt 11, 54 years, leiomyosarcoma) |
| 1.8 Abdominal pain |
| I had this strange stitch-like abdo pain. So that was kind of scary. I didn’t know if something was going on in my body that they could maybe be doing something about if I was at the hospital. (pt 6, 57 years, leiomyosarcoma) |
| I've got a big lump in my stomach, and occasionally that’s a bit sore and I get pain in my stomach. (pt 12, 54 years, leiomyosarcoma) |
| surgery was a relief from the symptoms [abdominal pains and discomfort] (pt 3, 61 years, undifferentiated spindle cell sarcoma) |
| **2. Sexual problems** |
| I haven’t had intercourse with my husband since the diagnosis (one year). I have an intense fear of causing vaginal bleeding again (pt 4, 58 years, rhabdomyosarcoma) |
| 2.1 Decreased ability to reach an orgasm |
| Well, after the first cancer the uterus was removed. And then I came here to the hospital to see a gynaecological oncologist. And then we spoke about the uterus and its function ‘Ah, you can do without it’. I said: ‘Well, you give up sexual enjoyment.’ And then he, that man, said: ‘Well, we investigated that at some point. But that is not the case’. I said: ‘I did. My orgasm really is much less.’ Because the uterus is a muscle. I had a big, nice, long lasting orgasm. And often. And that is gone. Just like that, gone together with the uterus. (pt 9, 59 years, leiomyosarcoma) |
| 2.2 Decreased interest in sex |
| sexual life is very limited now, vagina is dry and the uterus is removed (pt 2, age missing, leiomyosarcoma) |
| Because it's quite dry and it's sore I tried quite a bit of lubricants but that gets sore, so that does affect my libido. (pt 13, 56 years, endometrial stromal sarcoma) |
| To me, it’s been on my mind a lot. But before, you just have it regularly, when it comes instinctively when you’re in the mood. That is hardly there anymore. But I just know, that it always was really very good. And I want to keep it, or have it back. Women can get into the mood. I think. There have been times that we only [have sex] once in three months, well, I think that’s very serious. I think that’s a bad thing. I would prefer to have it once a week. But I don’t intrinsically want it. But I just still want to be able to do that. (pt 9, 59 years, leiomyosarcoma) |
| 2.3 Less sexual enjoyment |
| Then in 2013 I had a metastasis in the pubic bone. And then I had radiation therapy. So then everything down here got burned. Well, I thought that was also very serious, sexually. But it turned out okay. (pt 9, 59 years, leiomyosarcoma) |
| 2.4 Vaginal dryness |
| Because it's quite dry and it's sore I tried quite a bit of lubricants but that gets sore, so that does affect my libido. (pt 13, 56 years, endometrial stromal sarcoma) |
| **3. Gynecological problems** |
| 3.1 Heavy periods |
| Really heavy periods which was going on for, I think, sudden onset, they were getting heavier  (pt 13, 56 years, endometrial stromal sarcoma) |
| 3.2 Abnormal vaginal bleeding |
| So it kind of started with postmenopausal bleeding. (pt 6, 57 years, leiomyosarcoma) |
| The symptoms of the disease started when I noticed vaginal bleeding after intercourse with my husband (pt 4, 58 years, rhabdomyosarcoma) |
| I lost a lot of blood and there was, yeah, tissue with it. I was scared and then I went to see the GP and he sent me to the gynaecologist. (pt 10, 55 years, adenosarcoma) |
| Yes, in January last year I had lots of blood loss. So… And I thought: well, that may be menopause. But at some point I thought: that is more than what I had... I thought: it is not menopause. (pt 11, 54 years, leiomyosarcoma) |
| Because I have had quite a bit of blood loss. And then I really mean blood loss. So really like I was in a store and said: ‘What now?’ Like my blue jeans just turned red. Well, I experienced that three times. (pt 8, 59 years, leiomyosarcoma) |
| Very heavy bleedings, and I would just go to the hospital for some fibroids. (pt 9, 59 years, leiomyosarcoma) |
| **4. Urinary problems** |
| 4.1 Needed a catheter/ catheterising |
| I had a catheter for so long. (pt 9, 59 years, leiomyosarcoma) |
| 4.2 Urinary incontinence |
| Sometimes leak urine and have to rush sometimes to urinate (pt 3, 61 years, undifferentiated spindle cell sarcoma) |
| When I have to urinate, I have to go quickly. And it’s close. I wear incontinence products, so I won’t get into a situation where I pee my pants. But the incontinence product always seems like you don’t really need it. (pt 9, 59 years, leiomyosarcoma) |
| 4.3 Urinating frequently |
| but now the tumour is pushing on my bladder, I'm up in the night all the time going to the loo. (pt 12, 54 years, leiomyosarcoma) |
| I still need to urinate frequently (pt 3, 61 years, undifferentiated spindle cell sarcoma) |
| **5. Hormonal problems** |
| 5.2 Experiencing menopausal symptoms because I cannot take hormone replacement therapy |
| No, I was on HRT beforehand, and so, that was helping me with the hot flushes and everything, and then I had to stop taking it because of the cancer and everything. Then, the hot flushes came back. (pt 12, 54 years, leiomyosarcoma) |
| And obviously because my ovaries are removed and I'm not taking any hormones I am getting dryness and all this oestrogen deficiency symptoms. (pt 13, 56 years, endometrial stromal sarcoma) |
| **6. Neurological symptoms** |
| 6.1 Numbness |
| And then I get a numb feeling in my feet. (pt 9, 59 years, leiomyosarcoma) |
| 6.2 Tingling |
| Yes, I have a tingling feeling, when I am on the bicycle. (pt 9, 59 years, leiomyosarcoma) |
| After treatment a sort of tingling. So that is yet on nerve-level where you get an effect of, of that chemotherapy. (pt 11, 54 years, leiomyosarcoma) |
| **7. Functional impairment** |
| 7.1 Impairments in ADL |
| I need help. I can shower myself independently, but then my feet aren’t washed. And with dressing too. (pt 9, 59 years, leiomyosarcoma) |
| Yes, they all have to do more, because of course I don’t … Nothing anymore. Normally you did everything, keeping up with the household, but yes, you just do it… It is just not possible anymore. And the energy that you do have, you’re definitely not spending on that. (pt 10, 55 years, adenosarcoma) |
| I can't even go shopping because of infections (pt 2, age missing, leiomyosarcoma) |
| 7.2 Impairments in mobility |
| I couldn’t drive a car anymore. At the moment I still can’t drive. So the mobility, going, the freedom of going, the independence, is taken away. (pt 9, 59 years, leiomyosarcoma) |
| normally, I go off on big, long bike rides, and I can’t do that. […]I can’t cycle because I get too tired. (pt 6, 57 years, leiomyosarcoma) |
| 7.3 Physical impairment |
| I am not as fit as before (pt 1, 46 years, sarcoma NOS) |
| But during real exertion, then you of course feel that there still is a long way to go regarding stamina. (pt 11, 54 years, leiomyosarcoma) |
| And yes, walking the stairs or something, then.. Pff. Yes, then I first sit upstairs panting. (pt 10, 55 years, adenosarcoma) |
| I stayed at my mother’s last week and I used a set of stairs for the first time in months, and I found it really difficult. (pt 12, 54 years, leiomyosarcoma) |
| And on the other hand, in the past 1.5 years, I have been very much occupied with the fact that I can’t to run anymore, I can’t jump anymore. I was a gymnast. Yes, and I was always dancing. I still dance, but limited. (pt 9, 59 years, leiomyosarcoma) |
| But I do get a lot of backache, which is more soft tissue-related back ache if I do more lifting heavy things. But I can't do those type things and I avoid doing that as well, yes. (pt 13, 56 years, endometrial stromal sarcoma) |
| **8. Lack of energy** |
| 8.1 Feeling tired |
| the tiredness – I don’t have as much energy as normal, (pt 6, 57 years, leiomyosarcoma) |
| The other thing is I do feel tired, exhausted because of the oestrogen effect, I'm sure. (pt 13, 56 years, endometrial stromal sarcoma) |
| Sometimes I am so tired I have to lay in bed. (pt 2, age missing, leiomyosarcoma) |
| Initially after the operation I was tired (pt 3, 61 years, undifferentiated spindle cell sarcoma) |
| And now it is going alright, but I am just really tired. (pt 10, 55 years, adenosarcoma) |
| 8.2 Need to rest |
| I need to rest often (pt 1, 46 years, sarcoma NOS) |
| I do try to sleep more, because I know that that is one of the basic things that is important. But that is actually going well. And the thing is, when I’ve exert myself, then I notice that I need to recover a bit more. That I sleep more. (pt 11, 54 years, leiomyosarcoma) |
| And I do lie down for a bit at times or something. To see if I can close my eyes, if I can take a nap or something. To just save some energy. I notice that, now that I’m going into my fifth cycle, that it is accumulating. So it keeps getting heavier. (pt 8, 59 years, leiomyosarcoma) |
| 8.3 Feeling weak and lacking energy |
| I don’t have as much energy as normal (pt 6, 57 years, leiomyosarcoma) |
| I just don’t have that much energy. And I don’t want to lose it to things that are not necessary. So I take it easy. I go very slowly. I also don’t work then. I don’t babysit then. I actually don’t do anything that would somewhat cost energy. (pt 8, 59 years, leiomyosarcoma) |
| Well, I have decreased energy since the last surgery. (pt 9, 59 years, leiomyosarcoma) |
| I am just really weak. (pt 10, 55 years, adenosarcoma) |
| You got a couple of months where it’s just time, you can’t really do much, (pt 7, 39 years, endometrial stromal sarcoma) |
| I couldn’t do much at all. (pt 3, 61 years, undifferentiated spindle cell sarcoma) |
| **9. Trouble sleeping** |
| I don’t sleep well no. [..] I am also not very tired anymore, so I’m lying in bed because my body is tired.. By 12 o’clock or something I fall asleep again until 6 or 7 o’clock or something. So that’s allright, but it just takes long before you fall asleep. (pt 10, 55 years, adenosarcoma) |
| Sleeping very badly, that’s just very annoying. (pt 8, 59 years, leiomyosarcoma) |
| 9.1 Trouble sleeping because of having to go to bathroom frequently |
| I just haven’t slept since… God, I can't remember. But, I don’t know whether that’s to do with the menopause, because I wasn’t sleeping that well before, but now the tumour is pushing on my bladder, I'm up in the night all the time going to the loo. (pt 12, 54 years, leiomyosarcoma) |
| 9.2 Trouble sleeping because I worry |
| If I go to bed on time, at 10 PM, I don’t sleep and I am afraid of death. So I sleep badly. I go to bed when I feel tired. (pt 9, 59 years, leiomyosarcoma) |
| **10. Pain** |
| 10.1.1 Postoperative pain |
| Initially after the operation I was tired and in terms of pain, I experienced a lot of pain. (pt 3, 61 years, undifferentiated spindle cell sarcoma) |
| 10.1.2 Pain when lifting heavy things |
| But I do get a lot of backache, which is more soft tissue-related back ache if I do more lifting heavy things. But I can't do those type things and I avoid doing that as well, yes. (pt 13, 56 years, endometrial stromal sarcoma) |
| 10.2.1 Pain of the lump |
| I've got a big lump in my stomach, and occasionally that’s a bit sore (pt 12, 54 years, leiomyosarcoma) |
| 10.2.2 Pain in area of surgery |
| And now this operation so I do get pain along the abdominal adhesions, I guess. (pt 13, 56 years, endometrial stromal sarcoma) |
| 10.2.3 Back pain |
| I had backpain; it was kind of very strange low backpain [..]But when I spoke my doctor, she said it was probably the bone marrow injection that I’d given myself that had stimulated my bone marrow and caused some bone pain. (pt 6, 57 years, leiomyosarcoma) |
| I experienced pain in my back and leg (pt 3, 61 years, undifferentiated spindle cell sarcoma) |
| But I do get a lot of backache (pt 13, 56 years, endometrial stromal sarcoma) |
| 10.3 Need to take pain medication |
| I experienced a lot of pain and needed pain killers (pt 3, 61 years, undifferentiated spindle cell sarcoma) |
| **11. Radiotherapy related problems** |
| 11.1 Burning and painful skin |
| And then I got radiation therapy. So the whole thing gets burned. (pt 9, 59 years, leiomyosarcoma) |
| 11.2 Blisters of the skin |
| You get blisters. Red skin. And what can you do? And yes, no idea if the vagina on the inside also has blisters or… no idea. (pt 9, 59 years, leiomyosarcoma) |
| 10.3 Ulcer near the anus |
| The radiotherapy affected me negatively in the abdominal area. I suffered diarrhea and constipation and ulcer in the anus area.”(pt 4, 58 years, rhabdomyosarcoma) |
| **12. Chemotherapy related issues** |
| 12.1 Feeling ill due to chemotherapy |
| But then I was sick in bed for a week, I really was nauseous and I could only lay in bed. I really was sick and then we would slowly build up and then by the time you feel well you could go again. (pt 10, 55 years, adenosarcoma) |
| Headaches (pt 2, age missing, leiomyosarcoma) |
| I have 6 days during which I just don’t feel well. (pt 8, 59 years, leiomyosarcoma) |
| Then I had to throw up all the time and I was very sick, the first two times. And yes, afterwards I got less. In the sense that I felt less sick afterwards. It went well for the first two days and then after the third and fourth day I really got yes, I lacked appetite and I just felt bad. (pt 8, 59 years, leiomyosarcoma) |
| After my chemotherapy, I just had flu-like symptoms in a way, just a bit achy, [..]My throat was a bit sore, but I get that every time a few days after. (pt 12, 54 years, leiomyosarcoma) |
| 12.2 Lower resistance to infection |
| my immune system is weak (pt 2, age missing, leiomyosarcoma) |
| 12.3 Hair problems |
| my hair’s falling out. (pt 6, 57 years, leiomyosarcoma) |
| Obviously, my hair has started to fall out a bit, but I'm hoping that’s stopped now, you never know. (pt 12, 54 years, leiomyosarcoma) |
| I was affected by hair loss (pt 4, 58 years, rhabdomyosarcoma) |
| Yes well, so then the hair loss. Eyebrows, lashes. (pt 11, 54 years, leiomyosarcoma) |
| The colour of my hair has changed, there are white spots now instead of brown. (pt 2, age missing, leiomyosarcoma) |
| 12.4 Nail problems |
| my fingernails are dark too, they are black (pt 2, age missing, leiomyosarcoma) |
| My fingernails are struggling a little bit, so they’re getting a bit broken, (pt 6, 57 years, leiomyosarcoma) |
| 12.5 Eye problems |
| And at the end of each cycle, like in the third week, I get really sore eyes. So just all of a sudden, my eyes start streaming, and it’s like the tears are acid. (pt 6, 57 years, leiomyosarcoma) |
| 12.6 Mouth problems |
| I have mucositis in my mouth, this is disgusting, the taste is disgusting, there is no pain and it is not permanent, but it it is really burdening, nothing tastes anymore. (pt 2, age missing, leiomyosarcoma) |
| Then you just feel that food you know. You don’t taste the rest anymore, but you immediately get such a … So you think “well, I don’t taste anything, just that food.” It’s just not tasty. (pt 10, 55 years, adenosarcoma) |
| I feel that that liquid [chemotherapy infusion] just 6 days, that sounds very silly if I say so, but that has to do with my taste and everything. With that feeling like you’re nibbling on a nail. Not liking coffee and tea. Or not liking certain foods. Then I really feel like that liquid is bothering me. (pt 8, 59 years, leiomyosarcoma) |
| So that your saliva just gets dry. And that is still kind of… That actually is still the case. So when you start talking, you indeed notice that it is less. So saliva that is more dry. (pt 11, 54 years, leiomyosarcoma) |
| 12.7 Nose problems |
| The mucosa in the nose are decreased. (pt 2, age missing, leiomyosarcoma) |
| 12.8 Skin problems |
| I think my skin’s a bit drier than normal (pt 6, 57 years, leiomyosarcoma) |
| I have to scratch everywhere, there are even little wounds because of that (pt 2, age missing, leiomyosarcoma) |
| But I have gotten after that chemo after two weeks a kind of skin rash. (pt 11, 54 years, leiomyosarcoma) |
| my skin is darker. It looks as if I had been on vacation all the time (pt 2, age missing, leiomyosarcoma) |
| **13. Weight problems** |
| 13.1 Weight gain |
| Weight gain (pt 2, age missing, leiomyosarcoma) |
| And then doing athletics and music and still wanting to paint and sport and being slim. Unfortunately I am 15 kg more heavy than I ever was. (pt 9, 59 years, leiomyosarcoma) |
| 13.2 Weight loss |
| Weight loss (pt 2, age missing, leiomyosarcoma) |
| My appetite went down, I lost half a stone. (pt 3, 61 years, undifferentiated spindle cell sarcoma) |
| You gradually lose some weight. (pt 11, 54 years, leiomyosarcoma) |
| **14. Respiratory problems** |
| 14.1 Shortness of breath |
| I get a bit out of breath, but that’s a thing I've noticed more recently (pt 12, 54 years, leiomyosarcoma) |
| 14.2 Trouble breathing on exertion |
| The most intense thing I feel is maybe on exertion the pressure on my chest.[..] So I swam one kilometer the other day. And then I do feel the pressure on my chest and what not. (pt 11, 54 years, leiomyosarcoma) |
| **15. Symptoms related to metastasis or local treatment for metastasis** |
| 15.1 eating problems |
| eating is not joyful any more (pt 2, age missing, leiomyosarcoma) |
| I can't eat like before, only pudding and yoghurt, this is really shit (pt 1, 46 years, sarcoma NOS) |
| 15.2 mouth problems |
| My teeth have to be removed due to radiation [for jaw metastasis] (pt 1, 46 years, sarcoma NOS) |
| 15.3 problems with speaking |
| problems with talking (pt 1, 46 years, sarcoma NOS) |
| After the lung surgery [for lung metastasis] in 2012 I almost had no voice at all. And no support for my voice. (pt 9, 59 years, leiomyosarcoma) |
| **17. Surgery effects** |
| 17.2 Improvement of quality of life after treatment |
| Surgery was a relief from the symptoms I presented with [abdominal pains and discomfort] (pt 3, 61 years, undifferentiated spindle cell sarcoma) |

| **MENTAL HEALTH** |
| --- |
| **1.Fears/Worries** |
| 1.1 Scanxiety |
| Every time I go for a scan it is again the anxiety builds up until the diagnosis, I mean until the results come I am a bit anxious. That week is quite stressful going through the scans. And then I try to forget and get on with life and then closer to the date again the anxiety builds up. And I think most of the people would find that. (pt 13, 56 years, endometrial stromal sarcoma) |
| anxiety near the follow-up visit (pt 5, 46 years, PEComa) |
| 1.2 Fear of recurrence |
| But every day I am afraid that I am going to start feeling something related to cancer again. So there is always dust over the beautiful things. And that is very big. (pt 9, 59 years, leiomyosarcoma) |
| but it’s just understanding that it’s not something that in three years down the line they’ll just say, yes, you’re cured and you’re fine. But actually, this is a bit of a chronic… This is with me now forever. That’s the really hard part to wrap your head around and that’s making it quite hard to just deal with. (pt 7, 39 years, endometrial stromal sarcoma) |
| I felt a lump lower down so I returned to my GP who told me that it could be a fibroid or cancer. (pt 3, 61 years, undifferentiated spindle cell sarcoma) |
| And whatever that actually is, you can [think] a lot of things… What is that again? A growing tumour? Or is it a remnant of..? Or is it just because of the chemo? (pt 11, 54 years, leiomyosarcoma) |
| 1.3 Fear of death |
| I am all days, all night afraid of death. I never say it. I am saying it here now. Because it’s there. (pt 9, 59 years, leiomyosarcoma) |
| of me maybe not being around for much longer, or not seeing grandchildren. Yes, I think those are the things I find hard. (pt 6, 57 years, leiomyosarcoma) |
| I do get a bit upset sometimes when I think I can't see any future; I'm not going to see my niece get married. I’m getting a bit upset now, thinking about it, but that’s the only time I get upset. (pt 12, 54 years, leiomyosarcoma) |
| 1.4 Fear for treatment |
| because I was expecting to be sick all night and all that sort of thing, and I haven’t been sick. (pt 6, 57 years, leiomyosarcoma) |
| Yes, I’m not waiting for it.[..] You just know that you’re going to be sick those 6 days. That’s not nice. (pt 8, 59 years, leiomyosarcoma) |
| 1.5 fears about having sex |
| I have an intense fear of causing vaginal bleeding again, and am embarrassed to discuss the issue with my physician. (pt 4, 58 years, rhabdomyosarcoma) |
| 1.6 Worried about your condition’s impact on loved ones |
| But I haven't discussed this with my family in [country of origin] because my family is all in [country of origin], my mother or my in-laws or my sister. But that's one thing we decided because they won't understand and if we have to just go for surveillance I think we will just leave it. So we haven't discussed it but quite a few of my friends here know about it. But in [country of origin] because they're far away we thought it best not to discuss and that's what I want. And I don't think it will make much difference. They would simply worry there. (pt 13, 56 years, endometrial stromal sarcoma) |
| Yes, probably mainly my children. So, yes, thinking about them and the impact on them is difficult. (pt 6, 57 years, leiomyosarcoma) |
| But my sons actually said, and especially the youngest... Of course it’s just a.. Yes, the news that that needs to happen, and he also went researching. And he came up with that cold cap, and he said: “if you go bald, I just can’t take that.”[..] and for those boys, and then you see it’s also just a process, it’s OK. Because if you do it for the first time, it’s all intense. You have chemo, and then your mother goes bald. And that is a lot at any age. It has quite an impact. And my youngest really struggled with it. So also the idea that I might go to his school, that you’ll have such a bald mother or whatever… All kinds of horror images. But that process of course, I am not alone in it. I am not doing it alone, but also with your family. (pt 11, 54 years, leiomyosarcoma) |
| My children were worried because they had recently lost their dad (pt 3, 61 years, undifferentiated spindle cell sarcoma) |
| Yes. I just said to [name physician] that you protect the people you love the most. At least I do. You don’t share it with them. While in general you might think that you can share everything with those around you. No. You protect them from your misery. Your young children want you to… “oh well, it’s going alright.” “Oh well, I am just a little tired.” You know? (pt 9, 59 years, leiomyosarcoma) |
| I’d say it’s more tough on my family. My family are finding it really difficult to take this in. (pt 12, 54 years, leiomyosarcoma) |
| And if people ask about it, then I can talk about it very well. But I struggle when people get sad about me. (pt 8, 59 years, leiomyosarcoma) |
| **2. Dislike of changed appearance / body image** |
| I am bothered by how I look (pt 1, 46 years, sarcoma NOS) |
| 2.1 Hair loss |
| I feel bothered because of the hair loss. (pt 2, age missing, leiomyosarcoma) |
| 2.3 Physical deformity |
| I find it bothering that people are staring at my face [because of jaw metastasis] (pt 1, 46 years, sarcoma NOS) |
| 2.4 Felt less attractive |
| And yes, are you attractive? No. (pt 9, 59 years, leiomyosarcoma) |
| **3. Felt constantly reminded about your condition** |
| Now it’s in my body in such a way, that you just notice with every step that you’ve been ill and have had cancer. I’ve really had it you know? So it’s always there now. And otherwise it was just there in your head still, but now it is here. (pt 9, 59 years, leiomyosarcoma) |
| but for the psychological aspect I still get post traumatic. [..]Yes, probably women come with heavy periods and [as their GP] I'm sending them for operation it just gets me the flashback of what I've gone through. Especially with women who are menopausal and I just try to… Don't take risks and I just send them as a fast track to be seen quickly. So a lot of things, the way I now at least I'm improving. Initially I couldn't even see any patients with heavy periods. I'm doing conception calls, I just couldn't do any of that initially, but now that gradually I've accepted and I've tried to move on. (pt 13, 56 years, endometrial stromal sarcoma) |
| **4. Living with uncertainty** |
| 4.1 Uncertainty regarding treatment |
| You can worry about it, but worrying about what will come, but not too seriously. So I can say like: “well, what will it do to me?” That you just don’t know very well. So the uncertainty. (pt 11, 54 years, leiomyosarcoma) |
| Yes. Well yes, yes. To the extent that it is effective, you also don’t know how long it will take before it comes back. (pt 8, 59 years, leiomyosarcoma) |
| 4.2 Uncertainty regarding the future |
| And my sense of security about your future, suddenly you’re busy with the disease again. (pt 9, 59 years, leiomyosarcoma) |
| And obviously, you know I’m only 39, I think just understanding what that means for your future life. I think that’s been the biggest blow for me and also because it’s not… It’s just so hard to digest but it’s also hard to know what questions to ask. (pt 7, 39 years, endometrial stromal sarcoma) |
| I do get a bit upset sometimes when I think I can't see any future; I'm not going to see my niece get married. I’m getting a bit upset now, thinking about it, but that’s the only time I get upset. (pt 12, 54 years, leiomyosarcoma) |
| Well, it’s more that you can’t look ahead. You have no prospect. It’s not like, it’s over next week or it will only get better. And that’s a bit what… Just not having prospect. [..] It’s waiting every time and, yes, I understand it though because they can’t do anything else. It’s just having no prospect, that’s it really. You’re sitting out your day, you’re sitting out your time. (pt 10, 55 years, adenosarcoma) |
| So it’s always a kind of uncertainty. So I just don’t know now. (pt 11, 54 years, leiomyosarcoma) |
| **5. Felt distressed by the lack of knowledge about sarcoma** |
| It’s just understanding the difference between sarcoma and the normal cancers, so to speak. That’s where, because it’s not really spoken about, it is not really understood in the same way as other cancers are. That’s making it quite hard. (pt 7, 39 years, endometrial stromal sarcoma) |
| So, yes, so I kind of was very much in the dark about what it all meant, what the treatment might be, and I very much wanted to find out. So I said to the consultant I didn’t even want to know where it was in my bones, because I didn’t want to worry. So I wanted to kind of just wait until I came to [sarcoma referral center] and saw [name physician] and she could explain to me exactly what was happening. So that three-week time was quite difficult, because I didn’t really know what was going on in my body, I didn’t know what I could do, I didn’t… I went to a spa for a weekend with my daughter, but I didn’t know if I could run or if I could swim, like if I did things that might mess it up or cause it to spread or something. I guess that was the scary thing. (pt 6, 57 years, leiomyosarcoma) |
| **6. Desire to have contact with fellow patients** |
| No. No. Well yes, the only thing, but that’s more what I’d have, but that’s also possible through an association, is that I also am very curious: how did other people experience this treatment? And how are they doing? But that is very personal of course. To have some sort of comparison: what’s the deal? What does that do to you? To compare a bit where you stand as well, or something. [..] somewhere you feel the need to sort of.. check. (pt 11, 54 years, leiomyosarcoma) |
| **7. Change in emotions** |
| I would say sometimes it’s more sad. (pt 12, 54 years, leiomyosarcoma) |
| Yes, I call it quiet sadness. [name physician] asked me: “what is quiet sadness?” Yes about what wishes you actually have. If that’s on your mind a lot, then you’ll get sad. So don’t do it. (pt 9, 59 years, leiomyosarcoma) |
| Sometimes, I have to say, the sadness comes up. [..] Yes, but I won’t sit crying all day. (pt 8, 59 years, leiomyosarcoma) |
| But I am just very tired and very emotional. (pt 10, 55 years, adenosarcoma) |
| **8. Felt stigmatized** |
| People talk about me because of my face [because of jaw metastasis] (pt 1, 46 years, sarcoma NOS) |
| **9. Shock of diagnosis** |
| I had a hysteroscopy. I was told then that it looked suspicious, so I prepared myself for needing a hysterectomy and assuming that I had cancer of the uterus, and then had quite a shock diagnosis when we went back to the hospital, when we found out it was in my liver, my lungs, my bone and the uterus and that it was this leiomyosarcoma, which I have never heard about before. (pt 6, 57 years, leiomyosarcoma) |
| I had zero symptoms; this was found out.[..] And as [the baby] was born, they found what they thought was just like a little nodule in my womb, which they said is probably nothing. Everyone has these, it’s kind of like endometriosis or something similar to that, nothing to worry about. Yada yada yada. And then we went through further research on it, it actually turned out it was sarcoma. It came completely out of the blue because at no point had I any kind of symptoms and actually the surgeon even said, unfortunately this type of sarcoma is often symptomless. If we wouldn’t have found it because of this, you would probably have found it two weeks later and there would be nothing I could do. (pt 7, 39 years, endometrial stromal sarcoma) |
| They did DNT like just hysteroscopy but they didn't pick up anything. I went back to the hospital and I had an emergency hysterectomy which all went fine. The doctor was good so everything and the result came it was quite shocking. So the diagnosis came after, after the operation, yes. (pt 13, 56 years, endometrial stromal sarcoma) |
| Then they removed my uterus. Because they thought there was a fibroid. So it wasn’t even a thought that there would be something malignant.[..] After the surgery I felt very well. Then I also felt like: ‘oh great.’ Something is removed from my body, that was bothering me. I really felt that at that time. After 6 days I got a phone call from the gynecologist, who said that alarming cells were found. Then I really felt like: ‘no, that’s not possible, because I feel so well. I am really doing well.’ But yes, then you get in such a rollercoaster of negative things. (pt 8, 59 years, leiomyosarcoma) |
| .. and I would just go to the hospital for some fibroids. And you think a short hospitalization. Well, in fact you’re still dealing with it[..] It was a huge shock. I couldn’t sleep that night. My son, my dearest, came to visit me and then you have to tell. Really, you just know that cancer is very bad. And you have it. And they removed 1,5kg from you. They didn’t say what kind of cancer. I was told in a very bad way. (pt 9, 59 years, leiomyosarcoma) |

| **SOCIAL HEALTH** |
| --- |
| **1. Loss of independence** |
| So I’ve become completely dependent on somebody close to me who wants to travel with me. And wants to carry my things. (pt 9, 59 years, leiomyosarcoma) |
| So, yes, I'm a bit worried about that, how I'm going to end up, or whether I should live with my family, or I might just save to move away. Luckily, I've got those options. I can do that financially, but I worry about whether I need to be near my family forever now, if you see what I mean. (pt 12, 54 years, leiomyosarcoma) |
| **2. Relationship with others** |
| 2.1 Losing contact with friends or family |
| my brother avoids me, he used to take me with him to parties because of my good looks, but now not any more [appearance changed because of jaw metastasis] (pt 1, 46 years, sarcoma NOS) |
| 2.2 Isolation |
| You don’t belong to the group of working people anymore. So you’re with the sick people. But I don’t have cancer at this time. Yesterday, there even was someone who said: ‘Are you going to work again?’ (pt 9, 59 years, leiomyosarcoma) |
| 2.3 Lack of understanding from others |
| But in that moment, like yesterday when I sat there with that doctor, I really felt like, he can act like I’m just healthy. Well, I’m totally not, definitely not. But I’m not sick, and I don’t feel sick. (pt 9, 59 years, leiomyosarcoma) |
| Because it’s got to stage four now, and I do accept it, but my family doesn’t. (pt 12, 54 years, leiomyosarcoma) |
| my close neighbour was shocked that I am strong and not afraid of the disease. (pt 4, 58 years, rhabdomyosarcoma) |
| **3. Limitation in activities** |
| 3.1 Limitation in social activities |
| not being able to participate in social events is a problem (pt 2, age missing, leiomyosarcoma) |
| I guess it’s more about how much has it stopped people being able to do things. So kind of, from my point of view, the sore eyes isn’t very socially nice, like if I’m going out and my eyes are bright-red and pouring tears. That’s quite difficult for a couple of days. But it’s not all the time. So that does impact on me. I would maybe think twice about meeting someone of an evening if that was the day my eyes seemed to be bright-red and pouring, just because it’s not very pleasant. (pt 6, 57 years, leiomyosarcoma) |
| During chemotherapy and because of treatment side effects I chose to lower my engagement with others, diarrhea and nausea were the main causes. (pt 4, 58 years, rhabdomyosarcoma) |
| Yes, I can’t manage to stay at a birthday party until midnight. I’ll have to pay for that. (pt 10, 55 years, adenosarcoma) |
| 3.2 Limitation in leisure activities or hobbies |
| No, I helped chaperone children at school during lunch [as a volunteer]. Well, that is just.. That just takes too much energy. (pt 10, 55 years, adenosarcoma) |
| Actually, I’d love to paint. And I have done some painting already, but the materials, the briefcase with paint, oil paint, that thing. I can’t bring on the bicycle. (pt 9, 59 years, leiomyosarcoma) |
| I can't do inline skating any more (pt 1, 46 years, sarcoma NOS) |
| And before, I used to do something like group lessons. Then I did some boxing and a bit of [specific type of] workout. With weights as well. But that was too much. And that is in a group as well. And I just didn’t want that anymore, because I didn’t want the group to fall behind, because I can’t keep up. And I also did bootcamp, but the running was too much. Then you’re falling behind from the group. So I didn’t want that. (pt 8, 59 years, leiomyosarcoma) |
| I can’t go swimming because of chemo, and I normally swim, like open water and swimming pools and things, quite a lot. So I can’t swim because of the chemo (pt 6, 57 years, leiomyosarcoma) |
| 3.3 Difficulty doing activities with (grand) children |
| Swimming is not possible because of infections, this is important because this is "quality time" with my daughter (pt 2, age missing, leiomyosarcoma) |
| Because when the other grandchildren come I can’t lift them. Or I’m holding them and I have no feeling in my right leg.[..] It’s different. I can’t walk with a child. What I would definitely enjoy, because you remember, it’s fun to walk with a child. Or dancing. No. (pt 9, 59 years, leiomyosarcoma) |
| **4. Financial difficulties because of medical costs** |
| I have a lot of financial problems due to the disease, I have far less money, only 250 euros for living (pt 1, 46 years, sarcoma NOS) |
| **5. Changes in work or study capacity** |
| 5.1 Had to change function at work |
| After the lung surgery [for lung metastasis] in 2012 I almost had no voice at all. And no support for my voice. I always sang. I was a music-teacher. [..] So I started to teach fulltime. Not as a music-teacher, but just a regular class. (pt 9, 59 years, leiomyosarcoma) |
| 5.2 Limitations in work/study |
| I stopped that study for a little while. So I’ve really been busy trying to get myself through this. And I had the opportunity to do so. (pt 11, 54 years, leiomyosarcoma) |
| No, but that’s mostly because I would have to start at 6 AM, and I think that’s just really..[..] Yes, then I’m making my night’s rest just too short. I already don’t sleep very well. So I already wake up a few times. (pt 8, 59 years, leiomyosarcoma) |
| I reduced the workload, I used to do a lot of out of hours and all, I've reduced that. (pt 13, 56 years, endometrial stromal sarcoma) |
| 5.3 Not being able to work |
| I haven’t worked properly since December, because I was off for three months after my hysterectomy, and as the sarcoma was diagnosed, I just… As I said, I returned for literally two weeks, and then I had to start treatment. (pt 12, 54 years, leiomyosarcoma) |
| Physically, at that time, I couldn’t work (pt 3, 61 years, undifferentiated spindle cell sarcoma) |
| And the occupational therapist said: ‘What do you still want to do [regarding work] with leiomyosarcoma? Really a very bad prognosis.’ [..] I can’t just lift a child. I can’t lift anymore. I often forget that. So I also had to give up my work[ as a teacher]. Well, that’s actually very sad as well. (pt 9, 59 years, leiomyosarcoma) |
| I can't work any more, I am retired, miss my job (pt 1, 46 years, sarcoma NOS) |
| And it was a big part of my life, and I did really love being a [..] lecturer, so I really liked my job. Yes, and, also, I think it’s kind of a shame that I’m now really good at that job, like all the years that I’ve invested in it, now I’ve got such a lot to give, it seems a bit of a waste of my talents not to use it. (pt 6, 57 years, leiomyosarcoma) |
| boredom, because I can not do my job anymore because of possible infections (pt 2, age missing, leiomyosarcoma) |
| **6. Having a changed life** |
| It has massive implications and it’s made a huge difference to me, but it’s almost like, if we have this conversation four years down the line, I can look back and say, this is what changed. (pt 7, 39 years, endometrial stromal sarcoma) |
| everything has changed (pt 2, age missing, leiomyosarcoma) |
| Because that’s just the way it is. So all of a sudden you’re on a different train. So you have to. And you can’t just do the things you did anymore. (pt 11, 54 years, leiomyosarcoma) |
| And also that I enjoy the peace every day. Because I’ve had a very hectic life. You’d be getting up at 6.30AM, laundry in the dryer, washing machine on, dryer on, making sandwiches, eating. And then at 7.45AM I’d bes taking the chairs from the tables at school and preparing and then having coffee and coming home at 6PM. And then doing athletics, and music and wanting to paint, to do sports, and be slim. [..] Unfortunately I am 15 kg heavier than I ever was. But well, I get up at 9 AM, I take a shower, it’s 10AM. I drink coffee. I live very slowly. And nice. (pt 9, 59 years, leiomyosarcoma) |
| it’s not likely that I’m going to go back to work, anyway. So, yes, I’ve kind of come to the point, I’m 57, I want to just spend more time at home and enjoy the garden and do the things I’m doing. (pt 6, 57 years, leiomyosarcoma) |

ADL: activities of daily living

**HCP quotes**

| **PHYSICAL HEALTH** |
| --- |
| **1. Gastrointestinal symptoms** |
| Yes. I mean they usually have all these abdominal issues, abdominal pain. It can be constipation, it can be diarrhoea on the other hand due to the different surgeries they had. (HCP 13, 45 years, medical oncologist) |
| 1.2 Heaviness abdomen |
| She has uterine… Low grade intrauterine stromal sarcoma. Of course, they come with the vaginal discharges and all of those things, and the heaviness lower abdomen. Heaviness and pain and all of those things. (HCP 2, age missing, medical oncologist) |
| 1.3 Swelling of the abdomen |
| And a bloated feeling or is your abdomen just swollen. [..] I saw a woman yesterday, who had a recurrence in her lower abdomen, who said to me: ‘I can tie my shoelaces again. I can do it myself again.’ Again the space that the tumour is taking up and then causing pain or the volume problem. (HCP 15, 58 years, medical oncologist) |
| 1.4 Problems with defecation |
| Because most of them have either an irresectable tumour and have [..] problems with defaecation. (HCP 15, 58 years, medical oncologist) |
| If there is a large sarcoma in the pelvic area, then you have potency disorders or bladder disorders or problems with defaecation. (HCP 7, 53 years, surgical oncologist) |
| Yes of course. After all these surgeries in the abdomen and gynaecological area, all these issues [regarding bowel movements or stools] are relevant. (HCP 13, 45 years, medical oncologist) |
| And then the other issues with regards to pelvic [?] abdominal [?] sarcomas are those issues related to how your bowel function is (HCP 3, 52 years, radiation oncologist) |
| 1.9 Rectal bleeding |
| Rectal bleeding (HCP 23, 45 years, nurse specialist) |
| **2. Sexual problems** |
| If you perform surgery around the bladder, prostate or in women down below, then that can definitely have sexual implications. (HCP 7, 53 years, surgical oncologist) |
| And of course it can be gynaecological sexual problems as well and things like that. (HCP 13, 45 years, medical oncologist) |
| Maybe their ability to continue normal sexual life probably. (HCP 1, 45 years, medical oncologist) |
| Then gynae, I suppose it’s sexuality as well as health symptoms as a consequence of just the surgery alone or surgery followed by other treatments. (HCP 17, 47 years, clinical oncologist) |
| 2.4 Vaginal dryness |
| That’s more a late effect of possible radiation therapy or an early effect from hormonal treatments. Actually, when you put it that way, you could say that a questionnaire completed by someone at six months after pelvic radiation, indeed would report a dry vagina. (HCP 10, 56 years, radiation oncologist) |
| They may have gone into surgical menopause so they may have vaginal dryness. (HCP 23, 45 years, nurse specialist) |
| 2.5 Vagina felt short or tight |
| Unless they specifically had a uterine sarcoma. (HCP 6, 41 years, surgical oncologist) |
| 2.6 Pain during sex |
| Yes, [pain during sex] indeed. But that’s more an anatomical problem. (HCP 23, 45 years, nurse specialist) |
| **3. Gynaecological symptoms** |
| 3.1 Heavy periods |
| Women who have get heavy periods and have fibroids can often be missed and end up having uterine leiomyosarcoma. (HCP 23, 45 years, nurse specialist) |
| 3.2 Abnormal vaginal bleeding |
| Was there a question about bleeding? Vaginal bleeding (HCP 23, 45 years, nurse specialist) |
| 3.3 Vaginal discharge |
| She has uterine… Low grade intrauterine stromal sarcoma. Of course, they come with the vaginal discharges and all of those things, and the heaviness lower abdomen. Heaviness and pain and all of those things. (HCP 2, age missing, medical oncologist) |
| **4. Urinary problems** |
| Because most of them have either a irresectable tumour and are in pain from it,problems with defaecation or passing urine. (HCP 15, 58 years, medical oncologist) |
| If there is a large sarcoma in the pelvic area, then you have potency disorders or bladder disorders or problems with defaecation. (HCP 7, 53 years, surgical oncologist) |
| And then the other issues with regards to pelvic or abdominal sarcomas are those issues related to how your bowel function is, how your bladder function is. (HCP 3, 52 years, radiation oncologist) |
| And I think it’s good to have the urinary ones in there, because sometimes, they may have had damage to pelvic floor or something like that. (HCP 18, 49 years, medical oncologist) |
| 4.2 Urgency to go to the toilet to urinate |
| Yes, it’s a good one, because if you have a tumour that is pushing on your bladder, your residual capacity is reduced. So then you have no bladder problem, but you need to urinate every half hour. (HCP 10, 56 years, radiation oncologist) |
| 4.4 Pain when passing urine |
| That won’t really occur, unless there is a bladder infection. (HCP 10, 56 years, radiation oncologist) |
| have you had pain or burning feeling when passing urine, yes a lot of them they come with this complaint (HCP 2, age missing, medical oncologist) |
| **5. Hormonal problems** |
| 5.1 Menopausal symptoms |
| about menopause symptoms if you lose your ovaries. If women have uterine leiomysoarcoma, especially if they are young women, if they are Estrogen Receptor positive you either suppress it or you have the ovaries removed if the disease is stimulated by ovarian activity and you put them in surgical menopause. Because they can’t have HRT, you have to be careful they don’t take it. They may further suppress it by putting them on anastrazol or letrozole. (HCP 23, 45 years, nurse specialist) |
| a gyne sarcoma patient, they may have had surgery that’s rendered them menopausal. So, they might have side effects from that point of view (HCP 18, 49 years, medical oncologist) |
| 5.2 No treatments possible for menopausal symptoms |
| For those patients, lots of those symptoms, you can’t treat them with HRT that you might do normally, and currently, we don’t have very good mechanisms for patients being seen. (HCP 18, 49 years, medical oncologist) |
| 5.3 Going into menopause early |
| I mean, it depends how old they are when they’re first diagnosed, but if the patients’ are diagnosed before the menopause, quite often, treatment will render them menopausal. I think rather than going in to a natural menopause, which, generally, is a slower decline of estrogen and progesterone, they go from having pre-menopausal levels to post very quickly. Sometimes, they can have much more pronounced symptoms. So, for those patients, it would be hot flushes, night sweats, insomnia, anxiety, sexual problems. (HCP 18, 49 years, medical oncologist) |
| It's not necessary to resect ovaries, resect the adnexes, but in many cases the surgeons take this decision. So, then the deprivation of steroids sometimes is also important in some cases, due to this surgery. (HCP 4, 55 years, medical oncologist) |
| 5.4 Pain in muscles and joints |
| Pain in muscles or joints is related to hormonal treatment that we rarely give. (HCP 15, 58 years, medical oncologist) |
| They mentioned about pain in the joints. So, some of the drugs that we use can cause pain in the joints [..]I think they’re probably talking about, there’s some drugs called aromatase inhibitors, so they were developed for the treatment of breast cancer, but they’re also used for treating of gyne malignancies. (HCP 18, 49 years, medical oncologist) |
| **6. Neurological symptoms** |
| 6.2 Tingling in feet |
| [Tingling in] hands I can’t really relate that to a pelvic problem, but feet can. (HCP 10, 56 years, radiation oncologist) |
| Because of nerve compression in the area tingling or numbness of your feet. (HCP 11, 35 years, medical oncologist) |
| **7. Functional impairment** |
| 7.1 Impairment in ADL (Needed help with ADL) |
| Yes I think more than other tumour types, I’ve seen patients, especially if they’ve had femoral nerve injury, femoral nerve resections or big pelvic resections. The patients require at least in the short term, maybe in the long-term help with IADLs and ADLs as well. (HCP 6, 41 years, surgical oncologist) |
| 7.2 Impairment in mobility |
| she cannot walk anymore because we resected the sciatic nerve, some root of the sciatic nerve, and she is in a wheelchair. (HCP 9, 44 years, surgical oncologist) |
| 7.3 Physical impairment |
| She was a runner, so she came to us because she was struggling to get back to running post-treatment. (HCP 21, 33 years, physiotherapist) |
| **10. Pain** |
| Because most of them have an irresectable tumour and particularly have pain from it. (HCP 15, 58 years, medical oncologist) |
| The tumour pain indeed (HCP 10, 56 years, radiation oncologist) |
| He had terrible tumour in the pelvis. He was going urine chemotherapy. She was in bed for many months with chronic pain therapy (HCP 9, 44 years, surgical oncologist) |
| pelvic or extremity sarcoma they can be in a lot of pain, pre-operatively. (HCP 6, 41 years, surgical oncologist) |
| She has uterine… Low grade intrauterine stromal sarcoma. Of course, they come with the vaginal discharges and all of those things, and the heaviness lower abdomen. Heaviness and pain and all of those things. (HCP 2, age missing, medical oncologist) |
| **13. Weight problems** |
| 13.1 Weight gain |
| Another this with menopause, is have they had weight gain? Many women feel that their body is changing, especially if they’re young. So there’s that. (HCP 23, 45 years, nurse specialist) |
| Weight gain, sometimes, people who do worry about them are gyne patients. (HCP 18, 49 years, medical oncologist) |
| 13.2 Weight loss |
| weight loss (HCP 23, 45 years, nurse specialist) |
| I think many patients with a concern about their weight around the time of diagnosis and they have difficulty gaining it back, and this can be distressing in itself. (HCP 6, 41 years, surgical oncologist) |
| **16. Local effects of tumour** |
| If you’ve got a gynaecological tumour, in the majority of cases, the site-specific symptoms are more because of the actual tumour rather than your treatment for it. (HCP 19, 37 years, medical oncologist) |
| 16.1 Fistulation |
| So, again, if they’ve had any fistulations or anything like that? Where faecal material might be coming out of their vaginal or urine might be coming out of their anus because this isn’t specifically post-op, so it could be that you have issues around fistulation (HCP 23, 45 years, nurse specialist) |
| 16.2 Mass protruding from vagina |
| She’s now got this massive pelvic tumour protruding out her vagina. (HCP 23, 45 years, nurse specialist) |
| 16.3 Deep venous thrombosis |
| Because a lot of them also, they come with the DVTs, those patients. (HCP 2, age missing, medical oncologist) |
| 16.4 Leg problems |
| [swelling of one or both legs] can indeed occur, congestion in the pelvic area. (HCP 10, 56 years, radiation oncologist) |
| The tumour can stop the whole, the lymph drainage. Or at least cut it off. (HCP 23, 45 years, nurse specialist) |
| **17. Surgery effects** |
| 17.1 Herniation |
| I guess questions about hernias or incisional issues long term because of the size and extent of the incisions. (HCP 6, 41 years, surgical oncologist) |
| 17.2 Improvement of quality of life after treatment |
| So I operated a patient three months ago, two months enough. She had terrible tumour in the pelvis. She was undergoing chemotherapy. She was in bed for many months with chronic pain therapy, and so we decided to go anyway to surgery even though progression of chemotherapy for a very, very high risk disease. And she finally had colostomy, then nephrostomy. She cannot walk anymore because we resected the sciatic nerve, some root of the sciatic nerve, and she is in a wheelchair. And I see her after two months of recovery in the rehabilitation ward and she was so happy. [..] But she was so happy because she had no pain like two months ago. So she said, thank you, thank you, I am really better than before. (HCP 9, 44 years, surgical oncologist) |
| The quality of life is improved enormously after the surgery. Because people get really sick from that sometimes, from those large tumours. Just literally get sick from it. And after the surgery they feel fitter and better and so on. (HCP 16, 42 years, surgical oncologist) |
| 17.3 Nephrostomy |
| So I operated a patient three months ago, two months enough. She had terrible tumour in the pelvis. She was undergoing chemotherapy. She was in bed for many months with chronic pain therapy, and so we decided to go anyway to surgery even though progression of chemotherapy for a very, very high risk disease. And she finally had colostomy, then nephrostomy. (HCP 9, 44 years, surgical oncologist) |
| 17.4 Swelling of legs |
| But [swelling of the legs] is usually something which is important after surgeries in that region. (HCP 13, 45 years, medical oncologist) |
| **18. Infertility** |
| Here the most frequent of course is the loss of potential to be… The loss of the capacity, ability to have child, to become pregnant of course. It's not necessarily to resect ovaries, resect the adnexes, but in many cases the surgeons take this decision. So, then the deprivation of steroids sometimes is also important in some cases, due to this surgery. (HCP 4, 55 years, medical oncologist) |
| especially pelvic surgery and radiation as well, is a very toxic chemotherapy, there is issues with fertility. (HCP 6, 41 years, surgical oncologist) |
| Or some patients with gynae sarcoma may be treated with hysterectomy and they for that become infertile. (HCP 1, 45 years, medical oncologist) |

| **MENTAL HEALTH** |
| --- |
| **1. Anxiety/Worries** |
| 1.1.3 Anxiety due to the delay of diagnosis and treatment |
| She has really struggled from a psychological point of view, partly because of earlier misdiagnosis, and she had that sort of protracted time to diagnosis and treatment. That’s brought with her a lot of anxiety, she’s had a change in career. She’s had a lot of psychological burden that she’s carried with her. (HCP 21, 33 years, physiotherapist) |
| 1.1.4 Anxiety after initial misdiagnosis |
| Women who get heavy periods and have fibroids can often be missed and end up having uterine leiomyosarcoma. So, that’s an issue because, I thought I have fibroids, and now I don’t. (HCP 23, 45 years, nurse specialist) |
| **2. Changed body image** |
| 2.4 Felt less attractive |
| If it’s a young woman with a gynaecological sarcoma, then sexuality could actually be a problem and that could have an impact on the question you’re asking [..]. In the sense of attractiveness. (HCP 10, 56 years, radiation oncologist) |
| do you feel less attractive? Of course, I think... Yes, I have never asked anybody about this, but I feel they got affect. (HCP 2, age missing, medical oncologist) |
| And less feminine again I think that’s just suggested and that might cause people to worry. (HCP 20, 41 years, palliative care consultant) |
| 2.5 Felt less feminine |
| No, [feeling less feminine] only is applicable there where the location of the tumour is as such that it influences sexuality or fertility. (HCP 10, 56 years, radiation oncologist) |
| 2.6 Less self-esteem |
| because this isn’t specifically post-op, so it could be that you have issues around fistulation, menopause, weight loss, weight gain, self-esteem, do you feel attractive? That type of thing.  (HCP 23, 45 years, nurse specialist) |
| I think, probably, having a hysterectomy and if there were other surgery, can affect their perception of themselves or the feeling that something is missing. So, that can also have effects on the psyche, I think. (HCP 18, 49 years, medical oncologist) |
| 2.7 Felt body is changing |
| Many women feel that their body is changing, especially if they’re young. So there’s that  (HCP 23, 45 years, nurse specialist) |
| **10. Dealing with (risk of) infertility** |
| And then there was before the radiation there was this issue with fertility if there should be an operation, I think an colorectal operation to change her ovary location or not. And she already had a child but then there was the issue if you want to have another and she had to discuss the pros and cons with her husband and her family. And, yes, and now she’s okay with the decision. (HCP 12, 40 years, psychologist) |
| I think it’s very important in sarcoma, because the age of diagnosis is relatively higher in younger populations, so many, many young people get sarcoma relative as a proportion of people in sarcomas in general, so it is a disease of young adults and adolescents and especially pelvic surgery and radiation as well, is a very toxic chemotherapy, there is issues with fertility and we’ve had patients who have had to decide to end their pregnancy to get their cancer treatment. (HCP 6, 41 years, surgical oncologist) |
| So a young woman who still has her periods, who actually still wants to have children and then has a sarcoma in the lower abdomen, it’s a challenge to send someone to a fertility (outpatient) clinic for egg freezing and other preservation techniques that the gynecologist has, in the time that you have to arrange your treatment. Men have it much easier of course. (HCP 10, 56 years, radiation oncologist) |
| in women [fertility preservation] can delay [treatment] quite a bit. And that actually is a psychological burden, because ‘you say that I have a good prognosis, I want to have children, but I also want to be treated for this’. (HCP 10, 56 years, radiation oncologist). |
| **11. Lack of psychosexual support** |
| Gynae wise, in terms of, I suppose we don’t delve in that much as clinicians. I think psychosexual aspects are a big factor, would play a part, yes. (HCP 17, 47 years, clinical oncologist) |
| 11.1 Sexual problems are rarely discussed spontaneously |
| Honestly, I think that we never ask the questions about sex.[..] People also don’t mention it spontaneously. (HCP 15, 58 years, medical oncologist) |
| Not very often I think. Not as far as I ask, because so far how I worked was that I looked at what is the issue of the patient and that I do not had the structured interview with all these issues. So it’s seldom that they ask me to help them with sexual problems. (HCP 12, 40 years, psychologist) |
| Actually because we are a little bit conservative in Jordan. [..]Yes, it’s not almost, usually it’s not discussed, yes. (HCP 2, age missing, medical oncologist) |

| **SOCIAL HEALTH** |
| --- |
| **2. Relationship with others** |
| Yes. But [the impact of sexual problems on the relationship] is not talked about with enthusiasm. So you really have to go after it actively to find that out. (HCP 7, 53 years, surgical oncologist) |
| **3. Limitation in activities** |
| For the vaginal discharges because it’s like to be many vaginal discharges and it will be like excessive discharges coming. It’ll affect the patient’s life like going a lot to the bathroom for changes and those things. So, yes it is sometimes it will affect their time. Not always, but sometimes. (HCP 2, age missing, medical oncologist) |
| **5. Changes in work** |
| 5.1 Change in career |
| had a change in career. (HCP 21, 33 years, physiotherapist) |
| 5.2 Limitations in work |
| If you have frequent bowel movements or things like that then it may impact on your ability to work in particular environments, and then that may change what type of work that you can do and accept. (HCP 3, 52 years, radiation oncologist) |

ADL: activities of daily living
